# Comparative Functional Genomic Analysis of Two *Vibrio* Phages Reveals Complex Metabolic Interactions with the Host Cell

**DOI:** 10.3389/fmicb.2016.01807

**Published:** 2016-11-14

**Authors:** Dimitrios Skliros, Panos G. Kalatzis, Pantelis Katharios, Emmanouil Flemetakis

**Affiliations:** ^1^Laboratory of Molecular Biology, Department of Biotechnology, School of Food, Biotechnology and Development, Agricultural University of AthensAthens, Greece; ^2^Institute of Marine Biology, Biotechnology and Aquaculture, Hellenic Centre for Marine Research, HeraklionCrete, Greece; ^3^Marine Biological Section, University of CopenhagenHelsingør, Denmark

**Keywords:** bacteriophages (phages), phage therapy, *Vibrio*, sirtuins, comparative genomics, phage–host interaction, nucleotide metabolism, NAD^+^-dependent deacetylation

## Abstract

Sequencing and annotation was performed for two large double stranded DNA bacteriophages, *φ*Grn1 and *φ*St2 of the *Myoviridae* family, considered to be of great interest for phage therapy against *Vibrios* in aquaculture live feeds. In addition, phage–host metabolic interactions and exploitation was studied by transcript profiling of selected viral and host genes. Comparative genomic analysis with other large *Vibrio* phages was also performed to establish the presence and location of homing endonucleases highlighting distinct features for both phages. Phylogenetic analysis revealed that they belong to the “schizoT4like” clade. Although many reports of newly sequenced viruses have provided a large set of information, basic research related to the shift of the bacterial metabolism during infection remains stagnant. The function of many viral protein products in the process of infection is still unknown. Genome annotation identified the presence of several viral open reading frames (ORFs) participating in metabolism, including a Sir2/cobB (sirtuin) protein and a number of genes involved in auxiliary NAD^+^ and nucleotide biosynthesis, necessary for phage DNA replication. Key genes were subsequently selected for detail study of their expression levels during infection. This work suggests a complex metabolic interaction and exploitation of the host metabolic pathways and biochemical processes, including a possible post-translational protein modification, by the virus during infection.

## Introduction

The ever growing demand for fishery products and seafood has led to intensification of aquaculture. The overuse and abuse of antibiotics has resulted in the selection of resistant bacteria that are considered one of the biggest risk for humanity. Bacteriophage therapy has been suggested as a potential alternative method for both treatment and prophylaxis of bacterial infections, including aquaculture, showing very promising results (Stone, 2002; Sulakvelidze, 2011; Jassim and Limoges, 2014). The advancement of sequencing technology has boosted the genomic characterization of isolated phages providing fascinating insights to their biology and interaction with their host. Furthermore, genomic analysis may provide crucial information for their safety as potential therapeutics as it is of imperative importance to exclude temperate phages or phages carrying toxin or antibiotic resistance genes (Muniesa et al., 2004; Modi et al., 2013).

Although the T4 bacteriophage’s genome has been fully sequenced, annotated, and characterized for many years now (Miller et al., 2003b), the need for T4-like phages genomic characterization from a varied host range remains high, with the number of newly isolated bacteriophages and their genomic characterization increasing (Klumpp et al., 2012).

By using protein sequence similarity in the past, large *Vibrio* phages have been distinct from the T4-like phages. Known as “schizoT4like” they have been also proposed to be classified as KVP40-like phages (Lavigne et al., 2009) mainly because of the size of the head and the high genome and protein similarities with the KVP40 phage (Miller et al., 2003a). According to GenBank this group now contains the *Vibrio* phages KVP40, *φ*pp2 and nt-1 all of which seem to be both morphologically and genetically similar, with homing endonucleases (HEs), NAD^+^ biosynthesis and nucleotide metabolism related genes standing out.

HEs are enzymes found in all forms of microbial life, like phages or even mitochondria and chloroplasts of eukaryotes (Stoddard, 2014). HEs are known as transposable elements that prefer to duplicate into specific genomic regions, which can be either intron, intron-less, or intein sites by a phenomenon called homing (Dujon, 1989). *Seg*-like and *mob*-like phage HEs are known as free standing genes and they have been reported as responsible for horizontal gene transfer among host and phage neighboring genes (Zeng et al., 2009).

Additionally the presence of NAD^+^ biosynthesis and nucleotide metabolism related genes in bacteriophages have been mentioned in various works (Mesyanzhinov et al., 2002; Miller et al., 2003a; Petrov et al., 2010; Javed et al., 2014). By being obligate parasites, bacteriophages are known to carry genes toward their own benefit (Gazzaniga et al., 2009) for host metabolic manipulation. Sirtuins are able to deacetylase acetyl-lysine in proteins (Burgos et al., 2013) and their presence in large bacteriophage genomes is still enigmatic. It is natural for phages to carry molecular tools for metabolic reprogramming of their host, since they need them in order to replicate their genome, before capsid packaging.

Biological characteristics such as morphology, burst size, latency period, adsorption time, and *in vitro* and *in vivo* lytic efficacy have been previously reported for two isolated and partially characterized *Vibrio* phages. These *Vibrio alginolyticus* lytic phages have also been proposed as promising agents for disinfecting live feeds in aquaculture (Kalatzis et al., 2016), but information over genomic features was lacking. In this study, the phages were sequenced, annotated, and compared, revealing the largest known double stranded DNA *Vibrio* phage until now with various notable features. Combined study of presence and position of HEs provided insights, such as evolutionary relationships. We also attempted to describe metabolic interactions between the viral and the host biochemical processes by studying the relationship of presumable NAD^+^ increased biosynthesis, *Sir2* viral gene and the necessity of increased ATP accumulation for quality phage DNA replication.

Expanding the knowledge of genomic features and better understanding of the complex phage–host biochemical interactions can provide valuable insights for the efficient application of phage therapy.

## Materials and Methods

### Phages and Bacterial Host

Both phages *φ*Grn1 and *φ*St2 belong to *Myoviridae* family and have been isolated from coastal seawater in Crete, Greece (Kalatzis et al., 2016). The bacterial host was a clinical *V. alginolyticus* strain isolated from sick gilthead seabream (*Sparus aurata*) and has been fully sequenced (Castillo et al., 2015).

### Amplification, Precipitation, and DNA Extraction of Bacteriophages

Two liquid bacterial cultures of *V. alginolyticus* strain V1 in the exponential phase of growth were infected separately by bacteriophages *φ*St2 and *φ*Grn1. The infection was performed with a multiplicity of infection (MOI) of 10 and both tubes were incubated overnight at 25°C with reciprocal shaking. The following day, the cultures were centrifuged and their supernatants were filtered (0.22 μm), tittered and stored at 4°C. Having an optimal titer of 10^10^ PFU ml^-1^, phages were concentrated using a standard poly-ethylene glycol/NaCl precipitation (Supplemental Data Sheet S1). DNA extraction was conducted using a Qiagen protocol of the QIAamp DNA Blood Mini Kit (QIAGEN, Hilden, Germany) with the addition of ethanol 100% before the first column wash. A yield of at least 10 μg of DNA was retrieved. Finally, polymerase chain reaction (PCR) (Verity^TM^ Thermal Cycler, Thermo Fisher Scientific, Waltham, MA, USA) with 16S universal primers (forward: 5′-AGAGTTTGATCCTGGCTCAG-3′, reverse: 5′-GACGGGCGGTGTGTACAAG-3′) was conducted before and after DNase RQ1 (Promega, Madison, WI, USA) treatment in order to verify the absence of the host’s or other contaminant DNA (Turner et al., 1999; Glockner et al., 2000). DNA quality was evaluated with NanoDrop (Thermo Fisher Scientific, Waltham, MA, USA) measurements and agarose gel before library construction.

### DNA Sequencings and Annotations

Five micrograms of the extracted DNA were used for the construction of a pair-end library with an insert size of 800 bp following Illumina sequencing using an Illumina Hi Seq 2000 (Illumina, San Diego, CA, USA) sequencer. Sequencing was conducted at the Beijing Genomic Institute (Shenzhen, Guangdong, China) according to the manufacturer’s protocol. Possible contaminated reads, primers, N-terminus, and 3′-, 5′-low quality reads were trimmed off with an error rate threshold of 0.05. *De novo* assembly was conducted using Velvet software (Zerbino and Birney, 2008) under the Geneious platform (R8 version; Biomatters Ltd, Auckland, New Zealand). Finally, assembling resulted in single contigs in both occasions. Annotations were made using *ab initio* gene predictor Glimmer 3 (Delcher et al., 1999) and Rapid Annotation Subsystem Technology (R.A.S.T.; Aziz et al., 2008; Overbeek et al., 2014) where tRNAs were also identified. Hypothetical proteins were identified by using the B2Go (BioBam, Valencia, Spain) platform against non-reductant protein database and UniProt database with an E-value threshold of ≤10^-6^ which allowed to identify and manually annotate more coding DNA sequences (CDSs) for *φ*Grn1 and *φ*St2. Verification of tRNAs took place with tRNAscan-se software^1^ (version 1.21; Lowe and Eddy, 1997). Synteny was studied by using MAUVE software (Darling et al., 2004) and a list of high homologous genes (blastp threshold: 90) was generated with the online software CoreGene (Zafar et al., 2002). Kyoto Encyclopedia of Genes and Genomes^2^ (K.E.G.G.; Ogata et al., 1999; Kanehisa et al., 2016) was used for verification of protein products involved in the metabolic processes described in the study.

### Whole Genome Alignment and Phylogeny

Whole genome alignment was carried out using the LastZ algorithm (Harris and Pierpoint, 2012). Similarity between the phages was recorded as the highest identity of distances between the different alignments. Whole genome neighbor-joining consensus tree with free end gaps and Tamura–Nei method (bootstrap: 10, consensus method threshold: 87%) was generated, after the alignment, with Geneious (Biomatters Ltd, New Zealand) software. A maximum-likelihood phylogenetic tree for HEs was generated with the MEGA 6 software (Tamura et al., 2013), Jones–Taylor–Thornton substitution model and nearest-neighbor-interchange model of tree interference (100 bootstrap). DELTA-BLAST algorithm was used to compare, identify, and characterize HEs amino acid sequencings and domains. Visualization of alignments was performed with the BioEdit software (version 7.2.5) (Hall, 1999). Relative expression patterns were generated with SigmaPlot (Systat Software Inc., San Jose, CA, USA). Heat maps were created with the FiRe 2.2 Microsoft Excel add-on (University of Fribourg, Fribourg, Switzerland).

### Protein Structure and Modeling

The “beta-lactamase domain” open reading frame (ORF) was studied with InterProScan (Jones et al., 2014) and Prosite (de Castro et al., 2006) bioinformatics tools to explore putative domains and active sites. The viral Sir2/cobB protein was studied and visualized using the SwissPdb software viewer^3^ (version 4.1; Guex and Peitsch, 1997). Packing, solvent exposure, and stereochemical structure were evaluated with Verify 3D^4^ (Molecular Biology Institute, UCLA, Los Angeles, CA, USA; Bowie et al., 1991; Luthy et al., 1992) and Prosa II (Sippl, 1993; Wiederstein and Sippl, 2007). Investigation of zinc ligand in a finger-like binding site was conducted with ZincExplorer (Chen et al., 2013). Salt bridges were evaluated with ESBRI online software (Costantini et al., 2008).

### Transcriptional Study of Bacterial and Viral Genes

Gene expression (Supplemental Data Sheet 1) was studied in wild type (uninfected control) and phage-treated bacteria (MOI:100). Bacteriophages were incorporated into bacterial cultures during exponential phase. Three biological replicates were used for both treatments. After 1 min of vigorous shaking at 25°C, 5 ml of each phage-treated culture were harvested. Harvest was repeated at 5, 10, 20, and 30 min post-infection (p.i.). For the control treatments only one harvest per replicate was performed at 30 min. Cells were immediately centrifuged at 4°C and washed with 150 mM NaCl prior to RNA extraction. The duration of the experiment was set based on the latency time which is 30 min for both phages (Kalatzis et al., 2016).

RNA extraction was performed using a standard TRIzol^TM^ reagent (Thermo Fisher Scientific, Waltham, MA, USA) according to manufacturer protocol. This method resulted in at least 13 μg of RNA per sample. 6 μg of RNA per sample were treated with DNase RQ1 (Promega, Madison, WI, USA) according to manufacturer’s protocol. Samples were tested with PCR to verify purity from bacterial and viral DNA. RNA was then extracted by using a phenol:chloroform protocol. A 70% yield of RNA was retrieved after DNase treatment. Approximately 1 μg of RNA was used per cDNA synthesis by using Superscript II (Thermo Fisher Scientific, Waltham, MA, USA) enzyme according to manufacturer’s protocol. Both bacterial and viral primers for cDNA amplification were designed using Geneious software (Supplementary Table S1) and were tested against both genomic DNAs to confirm that a single amplicon of 70 bp would result from quantitative real-time PCR (qPCR). qPCR was performed on a StepOnePlus^TM^ Real-Time PCR System (Applied Biosystems, Foster City, CA, USA) using SYBR Select Master Mix (Applied Biosystems, Austin, TX, USA), gene-specific primers at a final concentration of 0.2 μM each, and 1 μl of the cDNA as template. Primer specificity and formation of primer dimers were monitored by dissociation curve analysis. The expression levels of *V. alginolyticus* gyrase A (*gyrA*) and the HSP70 protein (*dnaK*) were used as housekeeping (HK) genes to normalize cDNA templates. In order to evaluate the experiment’s and the HK gene’s reliability, two viral gene expression motifs were studied at first after normalization. The first one is a glutaredoxin gene (*grx*) which is considered as a late early transcribed phage gene and the second one the major capsid protein (*MCP*), which is considered as a late transcribed one (Luke et al., 2002; Supplementary Figure S1).

The GenBank accession numbers for these two new genomes correspond to KT919972 for *φ*Grn1 and KT919973 for *φ*St2.

## Results and Discussion

### Genome Sequencing and Annotation

**FIGURE 1:**
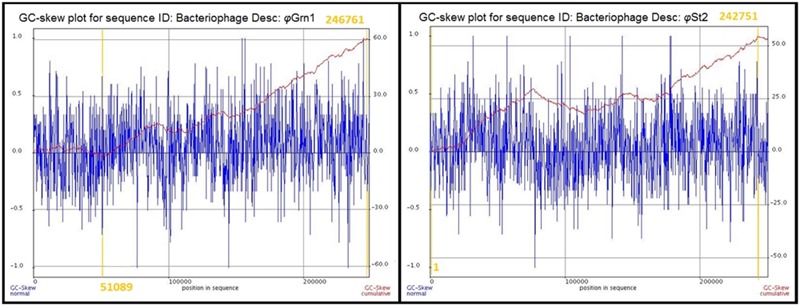
**Cumulative GC skews of phages *φ*Grn1 and *φ*St2.** The global minimum and maximum are displayed in the cumulative graph. Putative origin of replication and putative terminus location are highlighted.

The genomic sequences of the two bacteriophages were determined after the assembly of short raw reads. All nucleotides were aligned in one single contig of 248,605 bp and a GC content of 38.8% for *φ*Grn1 and 250,485 bp and a GC content of 42.6% for *φ*St2, which correspond to larger currently known *Vibrio* bacteriophage genomes according to GenBank. GC content of bacteriophages has been strongly linked to the host’s GC content in *Staphylococcus aureus* phages (Kwan et al., 2005), although disagreements have been mentioned in other species (Wittmann et al., 2014). The presented phages’ GC content is placed between other *Vibrio* phages like *φ*pp2 (42.55%) and KVP40 (42.6%) and *Enterobacteria* T4 phage (35.3%). Host’s GC content (*V. alginolyticus* V1 strain) is 44.5%. In order to identify the origin of replication and the terminus point of the phage genomes, cumulative GC skews were generated as described before (Grigoriev, 1998, 1999; Uchiyama et al., 2008; Jin et al., 2014). φGrn1 appears to have a putative origin of replication at 51,089 and a putative terminus location at 24,676, whereas *φ*St2 at nucleotide 1 and 242,751, respectively (**Figure 1**). Both phage GC skews agree with the transcriptional direction of most of the CDSs. Purine excess is strongly correlated with the leading transcriptional strand (Freeman, 1998).

In total, 410 and 412 genes were annotated for *φ*Grn1 and *φ*St2 phages, respectively (Supplementary Tables S2 and S3; Supplementary Figures S2 and S3). By using the online software CoreGene, we were able to detect 77 proteins with high identity among the T4 and large *Vibrio* phages contained in the GenBank; when we included only *Vibrio* “schizoT4like” phages in Coregene, identical proteins increased to 271 out of 381 of KVP40’s genome. This is strong indication that the newly characterized phages are “schizoT4like” and can been characterized as KVP40-like, and that VH7D should be included as well in that clade, increasing the number of known and characterized “schizoT4like” *Vibrio* phages from 3 to 6. When evaluating candidates for phage therapy, it is important to study thoroughly their genome for potential presence of known genes involved in bacterial resistance to antibiotics (Balcazar, 2014), especially when they are physically associated with transposable elements, like HEs. Comparative genome analysis revealed the presence of a small ORF in both studied genomes initially annotated as “beta-lactamase domain protein” (ALP47273 for *φ*Grn1 and ALP47653 for *φ*St2; Supplementary Tables S2 and S3). Interestingly a similar ORF is present in the KVP40 genome (NP_899337.1) and all *Vibrio* large phages cited in this work. Protein analysis and amino acid sequence comparison with characterized bacterial beta-lactamases, revealed that these polypeptides exhibited a low degree of similarity, while at the same time residue domains (data not shown) important for catalysis are absent. More specifically metallo-beta-lactamase domain, essential for catalysis (Moali et al., 2003), was not detected using InterProScan, while also no active site was detected using Prosite analysis. Finally, similarity with a well-known and characterized beta-lactamase of the Gram-negative bacteria *Stenotrophomonas maltophilia* (EC: 3.5.2.6) was lower than 1.4%. Thus, the results of these analyses do not support the automatic *in silico* annotation as this ORF does not appear to code for a functional beta lactamase protein. Further work is needed for the functional characterization of these ORFs, as the presence of active beta-lactamases on phages could represent a drawback for their application in phage therapy.

### Multiple tRNA Genes Are Present in Both Genomes

We verified the structure and the presence of tRNAs for *φ*Grn1 and *φ*St2, respectively, arranged in clusters in a region of approximately 10,000 bp (37,088 to 46,099) for *φ*Grn1 and approximately 8,000 bp (96,267 to 106,279) for *φ*St2. Both phages contained two pseudo-forms for GCA and TGC anticodons. Ten and seven hypothetical proteins are scattered inside the tRNA clusters of *φ*Grn1 and *φ*St2, respectively. tRNAs have only been found in double stranded DNA phages. The perception supported by T4 sequence that the average number of phage’s tRNAs was approximately 10 was challenged after the sequencing of many large phages, showing in addition that the presence of high number of tRNAs is a characteristic of virulent phages (Wilson, 1973); *φ*Grn1 a member of large phages presented here contains 28 (without pseudoforms), one of the highest number of tRNAs identified until today. Deletion of eight T4’s tRNAs has led to a decline in burst size and in phage protein synthesis, highlighting their significance and the evolutionary pressure that favors their conservation through natural selection (Freeman, 1998). Although phages harbor their own tRNAs, they are strongly host-dependent for the efficient translation of their proteins (Kunisawa, 2002). In our case, *V. alginolyticus* strain V1 possesses a number of at least 67 tRNAs. Codon usage of *φ*Grn1 is able to utilize its tRNAs and incorporate at least 41.8% of the amino acids in its proteins, while this percentage is 39.6% for *φ*St2. Codon usage from phage’s tRNAs is also associated with low and late expressed viral genes for which host’s tRNAs are not frequent (Kunisawa, 1992).

### Whole Genome Phylogenetic and Synteny Study

**FIGURE 2:**
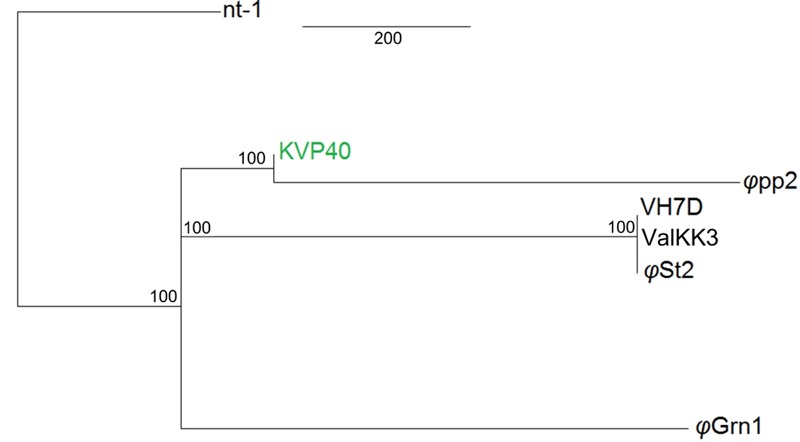
**Whole genome consensus tree.** Neighbor-joining consensus tree of all known *Vibrio* “schizoT4like” phages. Numbers next to branches represent consensus support. For improved visualization only closer whole genome alignments to the reference are shown. KVP40 bacteriophage was used as reference genome.

Both bacteriophages have the highest identity with two large *Vibrio* phages: ValKK3 (Lal et al., 2016; unclassified, possible “schizoT4like” *Vibrio* phage as well) and VH7D (Luo et al., 2015). Following whole genome alignment, identity was reported as the highest percentage between more similar alignments. The two phages had a 99.3% nucleotide identity. Highest genomic similarity was identified with VH7D (KC131129) reaching 98.74 and 99.24% for *φ*Grn1 and *φ*St2, respectively, with the ValKK3 (K671755) at 98.53%. Similarity with large phages KVP40 (AY283928), *φ*pp2 (JN849462), nt-1 (HQ317393), and reference phage T4 (AF138101) was also examined. *φ*Grn1 had a 94.13% similarity along KVP40 and a 94.5% with *φ*pp2, while *φ*St2 had 93.31 and 93.57%, respectively (**Figure 2**). Bacteriophage T4 having only a 168,903 bp genomic size, presents 55.99% similarity with *φ*Grn1 and 61.94% with *φ*St2.

**FIGURE 3:**
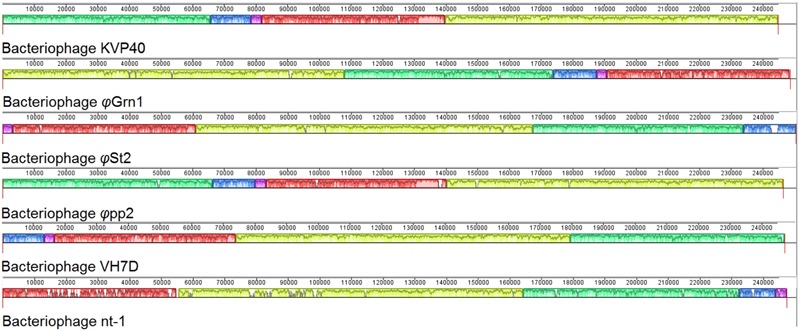
**Multiple genome alignment of bacteriophage genomes.** Genomes of all known *Vibrio* “schizoT4like” bacteriophages were compared using Mauve software. Local collinear blocks (LCBs) are highlighted with different colors. Same colored blocks indicate high synteny between genomes without genomic rearrangements. Graphs inside the blocks represent the level of synteny. White regions represent unique genomic regions. For improved visualization lines connecting the high syntenic regions have been obliterated.

Synteny of the genome organization between these phages was also examined as it has been proposed in *Acinetobacter* T4-like phages (Jin et al., 2014). The alignment of *φ*Grn1, *φ*St2, KVP40, *φ*pp2, nt-1, and VH7D resulted (**Figure 3**) into two small synteny local collinear blocks (LCBs) with 3,327 bp (purple) and 13,585 bp (blue) and three large LCBs with 57,120 bp (red), 66,636 bp (green), and 106,660 bp (yellow), indicating DNA regions which are homologous among the genomes. Graphs inside the blocks show very high similarity between the genomes, however, there are some non-identical genomic regions that are represented with white color inside the blocks. Although there seems to be a genomic rearrangement, the block sequence remains the same across the genomes of all phages, which can be speculated as a result of the possible circularly permuted linear double stranded DNA genomes of T4-like phages and their conserved genome organization (Petrov et al., 2006, 2010).

### Identification of Viral Lysozymes and Endolysins

Genome analysis also led to two ORFs for each phage belonging to the large lysozyme superfamily. The first one is a tail lysozyme at position 57,157 for *φ*Grn1 (ALP46994) and 116,942 for *φ*St2 (ALP47375). Both tail lysozymes contain the N-terminal of the OB domain, which was firstly thoroughly described for the T4 bacteriophage (gp5) (Nakagawa et al., 1985; Kanamaru et al., 1999; Arisaka et al., 2003). Additionally, one transglycosylase for each phage was detected at position 8,121 for *φ*Grn1 (ALP47078) and 69,098 for *φ*St2 (ALP47458). Both transglycosylases harbor the SLT domain characteristic of murein hydrolases (Van Asselt et al., 1999). Application of recombinant endolysins can be a major biotechnological weapon against infectious bacteria. Generally, difficulties toward successful endolysin utilization have been reported in Gram-negative bacteria, mainly due to their limited access to the interior peptidoglycan (Hermoso et al., 2007; Lai et al., 2011). However, application of endolysins in a slightly acidic environment has tackled this obstacle (Oliveira et al., 2014) and reports of successful extracellular applications of recombinant endolysins against Gram-negative bacteria have been recently reported (Lim et al., 2014; Oliveira et al., 2016).

### Unique HEs Are Present in Both Viral Genomes

**FIGURE 4:**
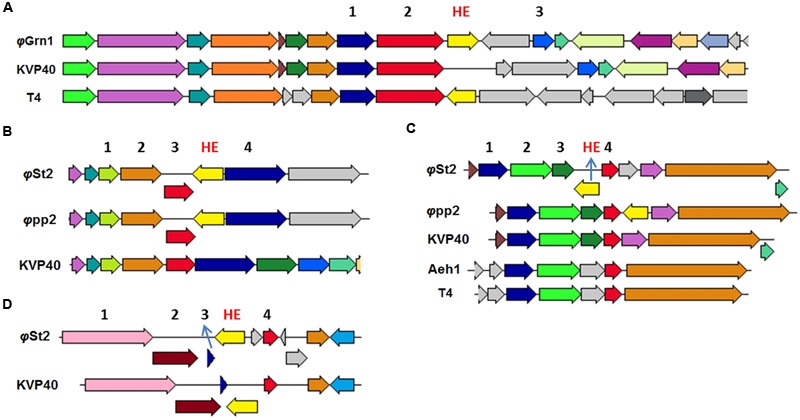
**Neighbor genes of HEs from various T4-like bacteriophages compared to *φ*Grn1 and *φ*St2.** Same colored arrows represent homologous genes. Light yellow arrows represent putative HE of compared bacteriophages. The genomic areas were aligned according to the gene represented from the red arrow. **(A)** Neighbor gene products of unique *φ*Grn1 *segD* HE. 1 for prohead assembly protein, 2 for major capsid protein, and 3 for inhibitor of prohead lysis. **(B)** Neighbor genes of *φ*St2 HE, *mob*-like. 2 for GTP cyclohydrolase I. 4 for hypothetical protein. **(C)** Neighbor genes of *φ*St2 unique HE, *seg*-like. 2 and 3 DNA polymerase clamp loader subunit. 4 for *regA*. **(D)** Neighbor genes of *φ*St2 *segD* HE. 1 for rIIA protector, 2 for rIIB protector, and 3 for hypothetical protein.

Both phages presented here bear HEs. With the increasing number of T4-like phages being sequenced and annotated, it becomes clear that the 15 HEs of the T4 phage is a feature unique to that phage (Edgell et al., 2010). Represented in our work, *φ*Grn1 has only one HE (like KVP40), while *φ*St2 has three (like *φ*pp2). We were able to identify conserved domains, compare and study syntenic relationships with other phage-associated HEs. The HE of *φ*Grn1 (ALP47050) is located at 90,429 and encodes for a 238 aa protein. It belongs to the *seg*-like HE family as it encodes the GIY_YIG domain of the homonymous superfamily. DELTA-BLAST showed a 43% similarity with *Salmonella* phage S16 HE (YP_007501215) with a 51% query coverage (E-value = 9 × e^-23^). Concerning *Vibrio* phages, ValKK3 presents the highest similarity with the *seg*-like HE (AJT61075) with a query coverage of 52% (E-value = 2 × e^-22^). BlastX algorithm could not match any nucleotide sequences with this particular HE. Synteny study showed that it is established at the same genomic area as the T4 phage *segD* HE (NP_049788.2), but with opposite orientation (**Figure 4A**). These data support the hypothesis that this is a unique HE, although the genomic region after the MCP is a frequent site of reported HEs in Enterobacteria phages (like T4). For *φ*St2, three HEs were identified and annotated. The first HE is a *mob*-like HE containing the HNHc domain (ALP47430) and is located at 240,309. It has a 98% similarity with the unique HE (Lin and Lin, 2012) of the *φ*pp2 bacteriophage (AFN37352) and a query coverage of 100% (E-value = 6 × e^-150^). It is also established at the same genomic area like *φ*pp2, revealing a possible evolutionary relationship between them (**Figure 4B**; Supplementary Figure S4). The other two HEs are characterized as *seg*-like endonucleases, because they both contain the GIY_YIG domain. Specifically, the second HE (ALP47453) at 216,143 appears to have only a 46% similarity with the HE of ValKK3 bacteriophage (AJT61075) with a query coverage of 97% (E-value = 3 × e^-43^) and subsequently should be enlisted as unique as well. Bacteriophage *φ*pp2 also has a HE established just after the *regA* gene, while it is located upstream in *φ*St2 (**Figure 4C**). Finally, the third HE (ALP47432) at 6,477 contains three main domains. Apart from the GIY_YIG domain, it also has two tandem repeats of the NUMOD3 domain (Sitbon and Pietrokovski, 2003) and presents the highest similarity (90% similarity with a 100% query coverage, E-value = 4 × e^-80^) with the *segD* HE of the KVP40 *Vibrio* phage (NP_899393), although it was previously described as a unique one. It is established at the same area as KVP40’s after the rIIA and rIIB early lysis protectors (**Figure 4D**; Supplementary Figure S4) suggesting an evolutionary relationship between the bacteriophages. In order to avoid deletion, transposition of HEs can take place within a phage genome without compromising the viability of the phage and can be responsible for genomic shuﬄing (Sandegren et al., 2005) because they can also cause mobility of the surrounding components (Belfort, 1991). Lin and Lin (2012) described the occurrence of unique HEs and their indels in bacteriophage *φ*pp2 as evidence of being a distinct new species different from KVP40 within the T4-like phage family. Although they act as selfish DNA features (Jin et al., 2014), reports and characterizations of viral HEs can highlight putative evolutionary processes. For instance, *φ*St2 although geographically distant, may be an evolutionary link between *φ*pp2 and KVP40. This can be also supported by the fact that *φ*St2 can also infect *V. parahaemolyticus* (strain V2; Kalatzis et al., 2016), which is also the host-species of *φ*pp2 and KVP40. Apart from the evolutionary impact that HEs have in phagic genomes and the population diversity that can confer (Lin and Lin, 2012), they are also responsible for regulating surrounding genes, highlighting the importance of locating and characterizing them in comparative genomic studies (Edgell et al., 2010; Stoddard, 2014).

### Viral Infection Results in Complex Metabolic Interactions with the Host

**FIGURE 5:**
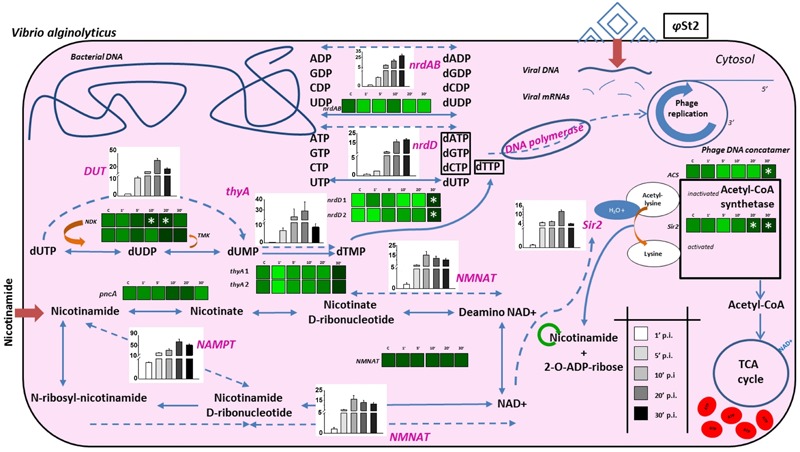
**Schematic representation of biochemical processes during the infection of *V. alginolyticus* V1 from bacteriophage *φ*St2.** Heat maps represent gradient changes in relative transcript levels of bacterial genes (black bolded) for control (C), 1 min post-infection (p.i.) (1′), 5 min p.i. (5′), 10 min p.i. (10′), 20 min p.i. (20′), and 30 min p.i. (30′) treatments. White asterisks represent statistically significant differences compared to control treatments (*p* < 0.05). Bars represent relative transcript levels of bacteriophage genes (purple bolded; ±SE) for 1 min p.i., 5 min p.i., 10 min p.i., 20 min p.i., 30 min p.i. treatments. Arrows represent bacterial processes and dotted arrows represent possible phage processes. Dark red arrows represent extracellular compounds that are inserted in the cell. Green cycle represents possible nicotinamide recycling.

The existence of NAD^+^ salvage enzymes in the genome of bacteriophage KVP40 has been previously described and thought to be of interest (Miller et al., 2003a). All of the *Vibrio* phages cited in the present publication bear a number of enzymes associated with the conversion of nicotinamide to NAD^+^, so it seems that large phages have the molecular tools to increase the cellular NAD^+^ content. Additionally, several bacteriophages carry genes with products involved in pyrimidine and purine biosynthesis. It is believed that large genome size imposes a strong need for DNA replication. Thus, the phage can increase the bacterial capacity for nucleotide biosynthesis in order to enhance the pathways and therefore its gene dosage (Karam and Drake, 1994; De Smet et al., 2016). This is also the case for the bacteriophages presented in this work. In the interest of pointing out the significance of the described genes and their effect during the infection, relative expression levels of both bacterial and bacteriophagic genes were studied (**Figure 5**; Supplementary Data Sheet S2; Supplementary Table S1), where the viral relative transcript levels outnumbered the host’s, as expected (Chevallereau et al., 2016). Apart from the nicotinamide mononucleotide adenyltransferase (*NMNAT*; ALP47012 for *φ*Grn1 and ALP47393 for *φ*St2), an enzyme present in both phages and the host, *φ*Grn1 and *φ*St2 also possess a nicotinamide phosphoribosyltransferase (*NAMPT*; ALP46980 for *φ*Grn1 and ALP47363 for *φ*St2), which is absent from *V. alginolyticus* and is able to utilize nicotinamide as a substrate and convert it to nicotinamide d-ribonucleotide, making a shortcut in the NAD^+^ biosynthesis pathway. No fluxes in the expression levels of the bacterial genes of pyrazinamidase (*pnac*) and *NMNAT* were noticed, showing that the pathway remains unaffected. On the other hand, *φ*St2’s *NAMPT* gene is transcribed almost immediately after the infection and *NMNAT* shows that the bacteriophage is probably utilizing intracellular nicotinamide instantly for NAD^+^ biosynthesis by using a quick two-step pathway. This suggests that bacteriophages try to enhance NAD^+^ production. Although many NAD^+^-dependent enzymes important for DNA replication have been reported (DNA ligase) in T4-like bacteriophages (Hertveldt et al., 2005), the characterized enzymes in the bacteriophages presented in this work are ATP-dependent or NADPH-dependent, except for a sirtuin, a deacetylase protein (Sir2/CobB protein, *Sir2*) (ALP47040 for *φ*Grn1 and ALP47418 for *φ*St2). This sirtuin is a NAD^+^-dependent conserved enzyme among large *Vibrio* phages and it was initially reported in bacteriophages after the sequencing of the KVP40 phage, where it was described as having a NAD^+^ hydrolysis role at the time. The homologous eukaryotic Sirt2 and Sirt3 proteins have been connected to increased lifespan and cell growth (Frye, 2000; Chang and Min, 2002). Both eukaryotic and prokaryotic sirtuins are known for post-translational modifications using NAD^+^ as a co-substrate. Specifically, deacetylation of acetyl-lysine by the sirtuins produces nicotinamide as a byproduct, which can also be recycled for NAD^+^ biosynthesis as mentioned above (Burgos et al., 2013). Deacetylation of acetyl-lysine has been strongly correlated with the activation of acetyl-coA synthetase (*ACS*) in prokaryotes, which is characterized as a Sir2-dependent enzyme (Starai, 2002). Lysine acetylation is a major post-translational modification in both prokaryotic and eukaryotic proteins and is a frequent regulatory phenomenon (Ouidir et al., 2015). *V. alginolyticus* strain V1 already possesses a Sir2/cobB deacetylase protein (*Sir2*). The presence of an additional highly divergent Sir2/cob protein in bacteriophages may suggest bacterial protein activation (such as the activation of ACS) by post-translational modifications by the virus. The strong relationship between *NAMPT* and sirtuins has been well described in prokaryotes and eukaryotes, along with the recycling of nicotinamide and the importance of the *de novo* synthesis of NAD^+^ (Imai et al., 2000; Lin et al., 2010; Burgos et al., 2013). The relative expression levels of the S*ir2* gene are high 20 min p.i., implying a possible priority of *NAMPT* and *NMNAT* regulation, and therefore NAD^+^ production before *Sir2* transcription. Sir2/cobB protein is able to activate ACS protein by deacetylation of acetyl-lysine and by consuming 1 ATP molecule, and therefore possibly advance toward the synthesis of acetyl-coenzyme A (AcoA; Gulick et al., 2003). Interestingly, a statistically significant increase in transcription levels is observed in the corresponding bacterial *Sir2* during late infection and in the *ACS* gene, which may be part of the phage’s metabolic manipulation and the biotic stress the cell is experiencing. AcoA can then be incorporated in the bacterial citric cycle and be one of the sources of increased intracellular ATP content by consuming the abundant NAD^+^. The presence of multiple ATP-dependent enzymes could indicate a high ATP demand during the lytic cycle. Direct evidence of increased accumulation of ATP during phage infection has been recently provided by Chevallereau et al. (2016; Supplementary Table S3). Additionally in our case, at least 97 genomes of 250,485 bp have to be synthetized during phage infection, which could dictate even higher nucleotide metabolic demand and subsequently energy in the form of ATP. Specifically, in association to purine metabolism, both phages carry the two subunits of a ribonucleoside diphosphate reductase (*nrdAB*; ALP46965, ALP46998 for *φ*Grn1 and ALP47345, ALP47379 for *φ*St2) and a ribonucleoside triphosphate reductase (*nrdD*; ALP46970 for *φ*Grn1 and ALP47350 for *φ*St2), both involved in the final steps of dATP and dGTP biosynthesis. In addition to *nrdAB* and *nrdD*, more enzymes are involved in pyrimidine metabolism; a dCMP deaminase (ALP47131 for *φ*Grn1 and ALP47515 for *φ*St2), a thymidine kinase (ALP47080 for *φ*Grn1 and ALP47460 for *φ*St2), and a thymidylate synthase (*thyA*; ALP47026 for *φ*Grn1 and ALP47405 for *φ*St2) are also present, enhancing dTTP biosynthesis from dCTP, and dUTP, a well-established fact during phage infection. Host and viral RNA decay could potentially be a source of free nucleoside diphosphates during T4-like phage infection (Carpousis et al., 1989, 1994; Ueno and Yonesaki, 2004; Uzan, 2009) and along with the presence of viral *nrdAB* and *nrdD* ATP-dependent ribonucleotidases, the phage might be able to enhance the much needed deoxyribonucleotide biosynthesis for DNA replication (Chevallereau et al., 2016). These enzymes reach their transcript levels plateau in 20 min p.i. for *φ*St2, while T4 bacteriophage—having a 20-min latency time—reaches these levels at 10 min p.i. (Luke et al., 2002). Statistically significant differences are noted in the two *nrdD* host genes, a phenomenon also observed recently during viral infection in a *Pseudomonas aeruginosa* strain (Chevallereau et al., 2016), an obligatory anaerobic enzyme. Bacterial ribonucleotide reductases are known for allosteric and transcriptional regulation depending on the balance of NTPs present in the cell (Torrents, 2014). Increased mutation rates during DNA replication can take place if uneven presence of NTPs is spotted (Wheeler et al., 2005). This upregulation of the two host *nrdD* reductases hint toward an imbalance of ATP content in the cell, which can be justified as described in this section. Interestingly, both phages contain a dUTP pyrophosphatase (*DUT*; AL47106 for *φ*Grn1 and AP47489 for *φ*St2), which has been reported in all *Vibrio* “schizoT4like” viruses and most bacterial species, but has not been reported or annotated in any *V. alginolyticus* bacterial strains (including V1). This host possibly lacks a dedicated enzyme for diphosphatase activity in order to one-step hydrolyze dUTP to dUMP, or that a possible chimeric protein has this enzymatic activity (Moroz et al., 2005). Nonetheless, *V. alginolyticus* can initially convert dUTP to dUDP with a nucleoside-diphosphate kinase (*NDK*) and then to dUMP with a dTMP kinase (*TMK*) (Kielley, 1970; Chakrabarty, 1998). DUTP pyrophosphatase has been extensively studied in yeast and proven to be efficient in preventing the incorporation of uracil into DNA during the replication stage (Gadsden et al., 1993). This suggests that whereas dUTP pyrophosphatase is absent in *V. alginolyticus*, lytic *Vibrio* bacteriophages carry it to possibly satisfy the need for quick uracil hydrolysis, which can interfere during the rolling circle replication if misused by DNA polymerase as a building block. This hypothesis is also supported by the presence of dUTPase in many retroviruses and the enzyme’s role in circumventing the deleterious effects of high uracil presence during the reverse transcription of the viral RNA (Hizi and Herzig, 2015). It is noteworthy that dUTP pyrophosphatase is found in most sequenced bacterial genomes, with *Escherichia coli* knock-out mutants resulting in accretion of putative short Okazaki fragments and subsequent errors in DNA replication (Tye and Lehman, 1977; Shlomai and Kornberg, 1978), while the T4 phage carries a bifunctional homologous dCTPase-dUTPase gene (gp56), which also takes part in forming 5-hydroxy-methyl-cytosine (Gary et al., 1998). Viral DNA replication peaks at 20 min p.i. (last one-third of the latency period), at the same time point that we were able to identify maximum expression levels for the *DUT* gene, showing a threefold increase in comparison to 10 min p.i. This indicates the possible need to shift the nucleotide biosynthesis balance toward DNA replication, rather than RNA production (transcription), in order to prevent DNA polymerase from using uracil as a substrate for DNA synthesis. At 10 min p.i. we also noticed fluxes in the transcription levels of the *NDK* (statistically significant) and *TMK* bacterial genes, possibly mirroring the high uracil content and the need to hydrolyze it. The importance of hydrolyzing dUTP and converting it to dTMP, and later dTTP, is also reflected by the presence of the *thyA* gene in the phage and its induced transcription levels, along with *DUT*. Fluxes of the two bacterial *thyAs* are also observed, but not statistically significant.

### *In silico* Functionality Study of Sir2/cobB Protein

**FIGURE 6:**
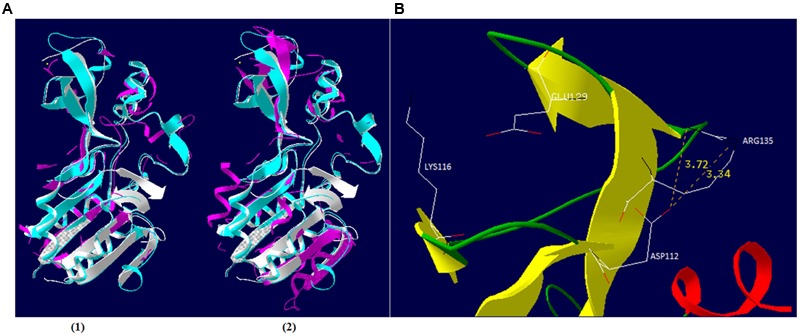
**Schematic representation of *φ*St2 and *φ*Grn1 Sir2/cobB protein. (A)** Similar (1) and dissimilar (2) amino acid residues of Sir2/cobB protein of *φ*St2 (magenta) are shown as superposition of structural models, with Sir2/cobB protein of *V. alginolyticus* (turquoise). Protein of *E. coli* is also shown (white). Ligand zinc of the finger domain is also represented (yellow sphere). **(B)** Ribbon diagram represents the finger domain of Sir2/cobB protein. Sheets (yellow), helices (red), and coils (green) are highlighted. Carbon (white), nitrogen (black), and oxygen (red) of the putative salt bridge residues are shown (represented as sticks). Distances between atoms are shown with yellow numbers (Armstrong).

**FIGURE 7:**
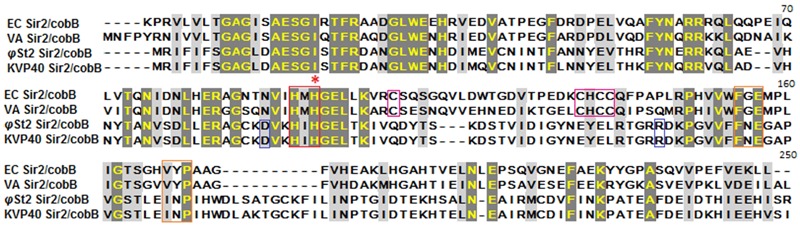
**Alignment of Sir2/cobB proteins of *E. coli, V. alginolyticus, φ*St2, and KVP40.** Red asterisk highlights the histidine active site. Boxes represent conserved regions of active sites (red), acetyl-lysine binding sites (orange), salt bridges of finger-like viral protein domains (blue), zinc ligand sites of finger-like bacterial protein domains (magenta).

In an attempt to provide insights in the relationship between NAD^+^ production and nucleotide biosynthesis, we noted the possible neuralgic role Sir2/cobB protein may have and tried to partially characterize it. Post-translational protein modifications are of high research interest, especially if they are taking place during a host–parasite interaction. Phages bear a large number of non-functional ORFs and studies aiming for functional verification can prove valuable. Both phages possess the same Sir2/cobB protein. A molecular model was constructed based on the Sir2/cobB crystal structure of the homologous protein from *E. coli* (PDB ID: 1SP5P; Zhao et al., 2004). Modeling predicted 10 protein sheets and 9 helices, along with a large Rossmann fold domain, a small Zinc binding domain, and the loops connecting the two (Supplementary Figure S5). Verify3D and PROSA II profiles (*Z* score -5.32) of packing, solvent exposure, and stereochemical structure proved that the final model was of high overall quality. Despite the low protein identity between *E. coli* and bacteriophage *φ*St2 (23.5 and 24.1% with *V. alginolyticus*), the overall predicted secondary structure is similar (**Figure 6A**). Unlike the bacterial protein, superposition of the phage’s protein shows absence of ligand zinc in the small finger domain, a phenomenon also observed in the Sir2/cobB protein of KVP40 bacteriophage. Furthermore, verification of the absence of a zinc ligand site was carried out by ZincExplorer software. This raises the question of functionality of the enzyme due to a possibly unstable secondary structure. Thus we examined the possibility of a salt bridge forming instead in the finger-like domain (Kumar and Nussinov, 2002). Although zinc binding sites can be extremely important for a stable secondary structure (Webster et al., 1991), salt bridges are able to substitute them efficiently in prokaryotic proteins (Baglivo et al., 2009). Prediction of salt bridges resulted in the detection of two possible sites between the aspartic acid and arginine residues in the 112 and 135 positions, respectively (**Figure 6B**) showing that the finger domain could remain stable. An additional salt bridge might also take place between lysine 116 and glutamic acid 129, with its presence needing further examination. Active site of the viral Sir2/cobB protein is detected at the histidine 112 position, a conserved region of bacterial and viral sirtuins. Additionally, the binding site of acetyl-lysine is also conserved, with domains FNE and INP creating a similar to *E. coli* tunnel for acetyl-lysine to bind close to the active site (**Figure 7**). Specifically, the strictly conserved phenylalanine 190 and proline 221 (of *E. coli* Sir2/cobB protein) are present, in contrary the bacterial conserved tyrosine 220 is replaced by the polarly neutral amino acid Asparagine. This is a somewhat usual replacement also present in at least Archaea *Archaeoglobus fulgidus’* Sir2Af1 (Ringel et al., 2014) and the human SIRT2 protein (Feldman et al., 2015), with the polarly neutral glutamine replacing tyrosine and remaining functional. Finally, valine 219 of *E. coli* is replaced with isoleucine, both being non-polar aliphatic amino acids. This information suggests that viral Sir2/cobB proteins of *Vibrio* “schizoT4like” phages can act similarly to the bacterial and are able to deacetylase acetyl-lysines of enzymes, like *ACS*, and subsequently activate them. The Sir2/cobB protein is also conserved at the genomes of *E. coli* bacteriophage T5 (Wang et al., 2005), at *Salmonella* phage SPC35 (Kim and Ryu, 2011), at *Cronobacter* phage vB_CsaM_GAP32 (Abbasifar et al., 2014), at *Pectobacter* phage My1 (Lee et al., 2012) and at *Klebsiella* phage JD001 (Cui et al., 2012).

## Conclusion

The knowledge of the genomic organization of bacteriophages provides a valuable insight into their interactions with the host cell and facilitates their efficient application for phage therapy. Although phage therapy is a relatively old technique, it still lacks in basic research in order to understand processes of auxiliary metabolism during infection. This work underlines the significance of clarifying biochemical processes and interactions during bacteriophage infection and also, host metabolic hijacking, as a result of features unmasked after DNA sequencing. By determining the genome of these two phages and describing genomic features we know six (possibly seven including ValKK3) fully characterized large “schizoT4like” *Vibrio* bacteriophages with a wide spectrum of bacterial hosts of *Vibrio* species, which threat fisheries and aquaculture, increasing our “armory” against vibriosis without the use of antibiotics. Both importance of detailed genomic study and characterization of endolysins as potential antibacterial agents are highlighted in the manuscript. Additionally, the characterization and localization of HEs resulted to the description of viral evolutionary relationships. In an attempt to expand our knowledge in phage infection and lysis efficacy, we monitored both bacterial and viral gene regulation related to NAD^+^ biosynthesis and nucleotide metabolism, during the latency period of the *φ*St2 phage. The results hint toward possible post-translational modifications by the viral *Sir2* gene in order to activate inert bacterial ACS protein, a binary model never previously described in detail. Although there is a high variability in the Sir2/cobB protein, its partial characterization indicates that it can act similarly to the bacterial ones and also contribute to the increased cell needs in ATP for an efficient phage DNA replication. Overall our data raise the possibility that the ability of large phages to maximize biochemical host exploitation could render phage therapy more efficient. Future experiments including the biochemical characterization of the Sir2/cobB protein, its deletion and the monitoring of intracellular ATP during large-size phage infection can strengthen our assumptions. This information could maybe apply in the future toward creating more efficient molecularly engineered virions in the battle against bacterial drug-resistant infections.

## Author Contributions

EF, PK, and DS conceived the study and designed the research. DS and PGK performed molecular work. DS performed bioinformatics work. DS and PGK analyzed the data. EF and DS wrote the manuscript and discussed the results and all the authors commented on the manuscript.

## Conflict of Interest Statement

The authors declare that the research was conducted in the absence of any commercial or financial relationships that could be construed as a potential conflict of interest.

## Footnotes

1 http://lowelab.ucsc.edu/tRNAscan-SE/

2 http://www.genome.jp/kegg/

3 http://www.expasy.org/spdbv/

4 http://services.mbi.ucla.edu/Verify_3D/
