# Supplementary material for: Comparative Functional Genomic Analysis of Two Vibrio Phages Reveals Complex Metabolic Interactions with the Host Cell
Source: Front Microbiol. 2016 Nov 14;7:1807. doi: 10.3389/fmicb.2016.01807 (PMC5107563; doi:10.3389/fmicb.2016.01807)
Supplement: Supplementary file 1 [file Table_1.PDF]

# Supplemental Table 1. Bacterial and viral primers, which were used for the relative expression gene study.

**Primers list**

| Gene | Forward/Reverse | Organism | Sequence | Gene | Forward/Reverse | Organism | Sequence |
|---|---|---|---|---|---|---|---|
| *MCP* | Forward | φSt2 | 5'-CGACCAATACGCAGTGAACG-3' | *dnaK* | Forward | *V. alginolyticus* | 5'-TCCTACACGTGTCTGCGAAA-3' |
| *MCP* | Reverse | φSt2 | 5'-CACACCTGCGTCCATTTCAG-3' | *dnaK* | Reverse | *V. alginolyticus* | 5'-CCGCCAGAAGCTTGGATAGT-3' |
| *grx* | Forward | φSt2 | 5'-TACGTGTGGTTGGTCTGGTG-3' | *gyrA* | Forward | *V. alginolyticus* | 5'-CGGTACTGAGCAGATCCCAG-3' |
| *grx* | Reverse | φSt2 | 5'-GCGTTCACCACAAACAGGAC-3' | *gyrA* | Reverse | *V. alginolyticus* | 5'-ACCAGAAGCACCGTTAACCA-3' |
| *DUT* | Forward | φSt2 | 5'-AGCTACAACCGGGTGATTCA-3' | *NMNAT* | Forward | *V. alginolyticus* | 5'-TGCAAAATGGTTGACGCGTT-3' |
| *DUT* | Reverse | φSt2 | 5'-TGTAGTGGGTGCGAACCAAT-3' | *NMNAT* | Reverse | *V. alginolyticus* | 5'-CTTGCTCTGCATCAGAACGC-3' |
| *Sir2* | Forward | φSt2 | 5'-TTTAGCGGTGCTGGTCTTGA-3' | *NDK* | Forward | *V. alginolyticus* | 5'-CTGGCCTACGTATCATCGCT-3' |
| *Sir2* | Reverse | φSt2 | 5'-TTTCCCACAGACCATTTGCG-3' | *NDK* | Reverse | *V. alginolyticus* | 5'-TCTGCATAAAAGCCGCTTGC-3' |
| *NMNAT* | Forward | φSt2 | 5'-TGCGATTCGTGAACTGCAAG-3' | *Sir2* | Forward | *V. alginolyticus* | 5'-ATGTAGCAACGCCTGAAGGG-3' |
| *NMNAT* | Reverse | φSt2 | 5'-TTTGAGAGAGCCACGCAAGA-3' | *Sir2* | Reverse | *V. alginolyticus* | 5'-TTGCGGCGTTGGTTGTAAAA-3' |
| *NAMPT* | Forward | φSt2 | 5'-TCGTGTCTCAACTGGTCGTT-3' | *pncA* | Forward | *V. alginolyticus* | 5'-CCAGCCAATATGCTTGAGCC-3' |
| *NAMPT* | Reverse | φSt2 | 5'-GTCAGGACGAGCAACAAACG-3' | *pncA* | Reverse | *V. alginolyticus* | 5'-CAAGCACACAGTGACGGTTC-3' |
| *nrdAB* | Forward | φSt2 | 5'-GCACAAGAAGCTGAACTGAGT-3' | *ACS* | Forward | *V. alginolyticus* | 5'-GATGAATCTTGCTGCTGCGT-3' |
| *nrdAB* | Reverse | φSt2 | 5'-TCAGGAATTCAGCGCGTACA-3' | *ACS* | Reverse | *V. alginolyticus* | 5'-GCCACATATCAACGCCGTTT-3' |
| *nrdD* | Forward | φSt2 | 5'-GCACAAGAAGCTGAACTGAGT-3' | *TMK* | Forward | *V. alginolyticus* | 5'-GCTAAAAGCGGCCGGTATTG-3' |
| *nrdD* | Reverse | φSt2 | 5'-TCAGGAATTCAGCGCGTACA-3' | *TMK* | Reverse | *V. alginolyticus* | 5'-TTTTTCTGCTAGCGGTGTGC-3' |
| *thyA* | Forward | φSt2 | 5'-AGCGGTTAATGAAGACCCGA-3' | *nrdD 1* | Forward | *V. alginolyticus* | 5'-ACGCGCTATTCGGTACAGAA-3' |
| *thyA* | Reverse | φSt2 | 5'-GAAGTACGGGTCCCATGCTT-3' | *nrdD 1* | Reverse | *V. alginolyticus* | 5'-ATGATCGCGACCGCTTTTTC-3' |
| | | | | *nrdD 2* | Forward | *V. alginolyticus* | 5'-TGTTAACGGACCGGGTACAC-3' |
| | | | | *nrdD 2* | Reverse | *V. alginolyticus* | 5'-ATAACAGCCACGGCACTGAT-3' |
| | | | | *thyA 1* | Forward | *V. alginolyticus* | 5'-TGACCGCGGTGAAATCTTGA-3' |
| | | | | *thyA 1* | Reverse | *V. alginolyticus* | 5'-CATACAAGGACGCAAGCACC-3' |
| | | | | *thyA 2* | Forward | *V. alginolyticus* | 5'-GCCAATGGGTATGGGAGTCA-3' |
| | | | | *thyA 2* | Reverse | *V. alginolyticus* | 5'-ATGGCGTAGTTTGTCGACCA-3' |
| | | | | *nrdB* | Forward | *V. alginolyticus* | 5'-AGACATCGCGCACTACTACG-3' |
| | | | | *nrdB* | Reverse | *V. alginolyticus* | 5'-CTCACCTTCACCGTAGCGAT-3' |
