# Supplementary material for: Comparative Functional Genomic Analysis of Two Vibrio Phages Reveals Complex Metabolic Interactions with the Host Cell
Source: Front Microbiol. 2016 Nov 14;7:1807. doi: 10.3389/fmicb.2016.01807 (PMC5107563; doi:10.3389/fmicb.2016.01807)
Supplement: Supplementary file 2 [file Table_2.PDF]

Supplemental Table 2. Gene products of *φ*Grn1 bacteriophage and their corresponding protein IDs. Last column represents presence (✓) or absence of homologous gene products in related published *Vibrio* “schizoT4like” bacteriophages (KVP40, *φ*pp2, nt-1, and VH7D) with an E-value threshold of 1.0 e^{-10}. Bold indicates *Sir2* gene and *italic bold* the unique homing endonuclease of the bacteriophage.

Supplemental Table 2. Gene functions of the *Vibrio* phage *φ*Grn1

Product	Start	Stop	nt (bp)	direction	protein id	Also present to			
						KVP40	*φ*pp2	nt-1	VH7D
Ribonucleoside diphosphate reductase alpha subunit	4386	6611	2226	forward	ALP46965	✓	✓	✓	✓
Ribonucleoside diphosphate reductase beta subunit	6621	7745	1125	forward	ALP46998	✓	✓	✓	✓
Thioredoxin	7747	8046	300	forward	ALP47203	✓	✓	✓	✓
Transglycosylase	8121	8693	573	forward	ALP47078	✓	✓	✓	✓
Thioredoxin	8980	9984	1005	forward	ALP47011	✓	✓	✓	✓
DNA topoisomerase	10030	11316	1287	forward	ALP46989	✓	✓	✓	✓
Queuosine biosynthesis QueE radical SAM	11567	12451	885	forward	ALP47028	✓	✓	✓	✓
Putative ATPase	15570	16274	705	forward	ALP47051	✓	✓	✓	✓
Anti-sigma factor	17553	17852	300	forward	ALP47202	✓	✓	✓	✓
Tail fibers protein	18817	23016	4200	reverse	ALP46957	✓	✓	✓	
Tail fibers protein	23087	23911	825	reverse	ALP47036	✓	✓	✓	
Tail fibers protein	28318	29856	1539	reverse	ALP46978	✓	✓	✓	✓
Putative ribonuclease with DUF458 domain	29924	30448	525	reverse	ALP47094	✓	✓	✓	✓
Deoxynucleotide monophosphate kinase	47493	48137	645	reverse	ALP47061	✓	✓	✓	✓
Tail completion protein	48367	48903	537	reverse	ALP47091	✓	✓	✓	✓
Baseplate hub assembly chaperone	51043	51891	849	reverse	ALP47032	✓	✓	✓	✓
Baseplate tail tube initiator	51904	52650	747	reverse	ALP47043	✓	✓	✓	✓
DNA end protector during packaging	52654	53250	597	reverse	ALP47070	✓	✓	✓	✓
Head completion protein	53643	54098	456	reverse	ALP47127	✓	✓	✓	
Baseplate tail tube cap	54167	55303	1137	forward	ALP46997	✓	✓	✓	✓
Baseplate wedge subunit	55300	55878	579	forward	ALP47075	✓	✓	✓	✓
Baseplate hub protein	55880	57151	1272	forward	ALP46991	✓	✓	✓	✓
Baseplate hub subunit/Tail lysozyme	57157	58368	1212	forward	ALP46994	✓	✓	✓	✓
Baseplate wedge subunit	60570	60989	420	forward	ALP47140	✓	✓	✓	✓
Baseplate wedge subunit (T4-like gp6)	61075	63033	1959	forward	ALP46969	✓	✓	✓	✓
Baseplate wedge subunit	63033	66530	3498	forward	ALP46959	✓	✓	✓	✓
Baseplate wedge subunit	66532	67554	1023	forward	ALP47008	✓	✓	✓	✓
Baseplate wedge tail fiber connector	67608	68564	957	forward	ALP47014	✓	✓	✓	✓
Baseplate wedge subunit and tail pin	68574	70820	2247	forward	ALP46963	✓	✓	✓	✓

Supplemental Table 2. Gene functions of the *Vibrio* phage φGrn1 *(Continued)*

Baseplate wedge subunit and tail pin	70820	71506	687	forward	ALP47054	✓	✓	✓	✓
Short tail fibers	71620	73044	1425	forward	ALP46985	✓	✓	✓	✓
Putative minor structural protein	73041	74468	1428	forward	ALP46984	✓	✓	✓	✓
Neck whiskers protein	74767	76449	1683	forward	ALP46974	✓	✓	✓	✓
Head completion neck hetero-dimeric protein	76460	77389	930	forward	ALP47018	✓	✓	✓	✓
Head completion neck hetero-dimeric protein	77393	78232	840	forward	ALP47034	✓	✓	✓	✓
Tail assembly protein	78344	79303	960	forward	ALP47013	✓	✓	✓	✓
Terminase small subunit	80015	80563	549	forward	ALP47086	✓	✓	✓	✓
Terminase large subunit	80523	82325	1803	forward	ALP46971	✓	✓	✓	✓
Tail sheath monomer	82372	84387	2016	forward	ALP46967	✓	✓	✓	✓
Tail tube monomer	84440	84940	501	forward	ALP47103	✓	✓	✓	✓
Portal vertex of the head	84980	86530	1551	forward	ALP46975	✓	✓	✓	✓
Prohead core protein	86544	86711	168	forward	ALP47323	✓	✓	✓	✓
Capsid and scaffold protein	86712	87203	492	forward	ALP47109	✓	✓	✓	✓
Prohead assembly (scaffolding) protein	87206	87847	642	forward	ALP47062	✓	✓	✓	✓
Prohead assembly(scaffolding) protein	87880	88728	849	forward	ALP47031	✓	✓	✓	✓
Major capsid protein	88799	90343	1545	forward	ALP46976	✓	✓	✓	✓
Homing endonuclease (Seg-like)	***90429***	***91145***	***717***	***forward***	***ALP47050***				
tRNA nucleotylditransferase	91194	92291	1098	reverse	ALP47001		✓	✓	
Inhibitor of prohead protease	92387	92884	498	forward	ALP47105	✓	✓	✓	✓
DNA helicase	98501	100033	1533	forward	ALP46979	✓	✓	✓	✓
Transamidase GatB domain protein	100520	100933	414	reverse	ALP47143	✓	✓	✓	
Single stranded DNA-binding protein	100930	101343	414	reverse	ALP47144	✓	✓	✓	✓
Tail connector protein	101512	104205	2694	reverse	ALP46961	✓	✓	✓	
Straight tail fiber	104214	107897	3684	reverse	ALP46961	✓	✓	✓	✓
Ribonuclease H	107976	108908	933	forward	ALP47017	✓	✓	✓	✓
Double-stranded DNA binding protein	108991	109272	282	forward	ALP47223	✓	✓	✓	✓
Transcriptional regulator	109256	109558	303	forward	ALP47199	✓	✓		✓
DNA helicase loader	109593	110153	561	forward	ALP47081	✓	✓	✓	✓
Single stranded DNA-binding protein	110204	111130	927	forward	ALP47019	✓	✓	✓	✓
Dihydrofolate reductase	111180	111725	546	forward	ALP47088	✓	✓	✓	✓
ATP-dependent Clp protease proteolytic subunit	111722	112438	717	forward	ALP47048	✓	✓	✓	✓

Supplemental Table 2. Gene functions of the *Vibrio* phage φGrn1 *(Continued)*

Recombination protein	112505	113605	1101	forward	ALP47000	✓	✓	✓	✓
DNA primase/helicase	114016	115299	1284	forward	ALP46990	✓	✓	✓	✓
Putative anaerobic ribonucleoside triphosphate reductase	115293	115538	246	forward	ALP47260	✓	✓	✓	✓
Ribonucleoside-triphosphtae reductase	115539	117374	1836	forward	ALP46970	✓	✓	✓	✓
Ribonucleoside-triphosphtae reductase activating protein	118491	118967	477	forward	ALP47116	✓	✓	✓	✓
Phosphoesterase	118967	119491	525	forward	ALP47093	✓	✓	✓	✓
DNA helicase	120405	121229	825	forward	ALP47037	✓	✓	✓	✓
DNA primase/ DNA helicase	121788	122846	1059	forward	ALP47003	✓	✓	✓	✓
Deoxyuridine 5'-triphosphate nucleotidohydrolase	122846	123343	498	forward	ALP47106	✓	✓	✓	✓
Exonuclease A	123582	124274	693	forward	ALP47053	✓	✓	✓	✓
Thymidylate synthase	126179	127078	900	forward	ALP47026	✓	✓	✓	✓
NAD-dependent protein deacetylase of SIR2/cobB family	130642	131403	762	forward	ALP47040	✓	✓	✓	✓
Topoisomerase IV subunit B	131538	133331	1794	forward	ALP46972	✓	✓	✓	✓
Ser/Thr protein phosphatase family protein	134715	135443	729	forward	ALP47046	✓	✓	✓	✓
Putative Hydrolase	136759	137352	594	forward	ALP47071	✓	✓	✓	✓
DNA ligase	137847	139184	1338	forward	ALP46988	✓	✓	✓	✓
RNA polymerase-ADP-ribosyltransferase Alt	140168	141709	1542	reverse	ALP46977	✓	✓	✓	✓
Glutaredoxin	143541	143780	239	forward	ALP47267	✓	✓	✓	✓
Capsid vertex protein	143836	144735	900	forward	ALP47025	✓	✓	✓	✓
RNA polymerase sigma factor	144744	145256	513	forward	ALP47096	✓	✓	✓	✓
Putative 5'(3')-deoxyribonucleotidase	149037	149537	501	forward	ALP47104	✓	✓	✓	✓
Recombination-related endonuclease	149534	150577	1044	forward	ALP47005	✓	✓	✓	✓
Recombination-related endonuclease	150794	153031	2238	forward	ALP46964	✓	✓	✓	✓
Sliding clamp DNA polymerase accessory protein	153793	154458	666	forward	ALP47058	✓	✓	✓	✓
Replication factor C small subunit / DNA polymerase clamp loader subunit	154525	155475	951	forward	ALP47058	✓	✓	✓	✓
DNA polymerase clamp loader subunit	155486	155977	492	forward	ALP47110	✓	✓	✓	✓
Endoribonulcease	156012	156392	381	forward	ALP47156	✓	✓	✓	✓
DNA polymerase	157110	159662	2553	forward	ALP46962	✓	✓	✓	✓
RNA ligase A	159985	161130	1146	forward	ALP46996	✓	✓	✓	✓
Beta lactamase domain protein	162526	162759	234	forward	ALP47273	✓	✓	✓	✓
3'-phosphatase 5'-polynucleotide kinase	162768	163685	918	forward	ALP47022	✓	✓	✓	✓
DCMP deaminase	178065	178517	453	forward	ALP47131	✓	✓		✓

Supplemental Table 2. Gene functions of the *Vibrio* phage φGrn1 *(Continued)*

NADPH-dependent 7-cyano-7-deazaguanine reductase	178572	179504	933	forward	ALP47016	✓	✓		✓
GTP cyclohydrolase I	179572	180240	669	forward	ALP47057	✓	✓	✓	✓
NADPH dependent preQ0 reductase	181650	182558	909	forward	ALP47024	✓	✓	✓	✓
Queuosine Biosynthesis QueC ATPase	182614	183330	717	forward	ALP47049	✓	✓	✓	✓
Head assembly chaperone protein	184726	185064	339	forward	ALP47177	✓	✓	✓	✓
Endonuclease	185485	185772	288	reverse	ALP47218	✓	✓	✓	
RNA ligase	186335	187342	1008	forward	ALP47010	✓	✓	✓	✓
DNA methyltransferase	188780	189358	579	forward	ALP47076	✓	✓	✓	✓
RIIA lysis inhibitor	190701	192770	2070	forward	ALP46966	✓	✓	✓	✓
RIIB lysis inhibitor	192763	193803	1041	forward	ALP47006	✓	✓	✓	✓
CAMP-dependent Kef-type K+ transport system	194565	195167	603	forward	ALP47069		✓	✓	✓
Chromosome segregation protein	199153	199422	270	forward	ALP47235	✓	✓	✓	✓
DNA helicase	199433	200698	1266	forward	ALP46992	✓	✓	✓	✓
Nicotinamide-nucleotide adenylyltransferase NadM family /ADP-ribose pyrophosphatase	203205	204230	1026	forward	ALP47007	✓	✓	✓	✓
Nicotinate-nucleotide adenylytransferase	204979	205515	537	forward	ALP47090	✓	✓	✓	✓
Thymidine kinase	218342	218911	570	forward	ALP47080	✓	✓		✓
Endonuclease V	226420	226824	405	forward	ALP47148	✓	✓	✓	✓
Nicotinamide-nucleotide adenylyltransferase NadR family/ Ribosylnicotinamide kinase	228909	229889	981	forward	ALP47012	✓	✓	✓	✓
Ribosyl nicotinamide transporter PnuC-like	230840	231514	675	forward	ALP47056	✓	✓	✓	✓
Adenylate cyclase	238503	239327	825	forward	ALP47035	✓	✓	✓	✓
Nicotinamide phosphoribosyltransferase	246974	248467	1494	forward	ALP46980	✓	✓	✓	✓
