# Supplementary material for: Comparative Functional Genomic Analysis of Two Vibrio Phages Reveals Complex Metabolic Interactions with the Host Cell
Source: Front Microbiol. 2016 Nov 14;7:1807. doi: 10.3389/fmicb.2016.01807 (PMC5107563; doi:10.3389/fmicb.2016.01807)
Supplement: Supplementary file 3 [file Table_3.PDF]

Supplemental Table 3. **Gene products of *φ*St2 bacteriophage and their corresponding protein IDs.** Last column represents presence (✓) or absence of homologous gene products in related published *Vibrio* "schizoT4like" bacteriophages (KVP40, *φ*pp2, nt-1, and VH7D) with an E-value threshold of 1.0 e^{-10}. **Bold** indicates *Sir2* gene and ***italic bold*** homing endonucleases of the bacteriophage.

Supplemental Table 3. **Gene functions of the *Vibrio* phage *φ*St2**

**Product**	**Start**	**Stop**	**nt (bp)**	**direction**	**protein_id**	**Also present to**			
						**KVP40**	***φ*pp2**	**nt-1**	**VH7D**
RIIA lysis inhibitor	3229	5298	2070	forward	ALP47346	✓	✓	✓	✓
RIIB lysis inhibitor	5291	6274	984	forward	ALP47392	✓	✓	✓	✓
Homing endonuclease (segD)	***6477***	***7172***	***696***	***reverse***	***ALP47432***	✓			
Chromosome segregation protein	13140	13409	270	forward	ALP47373	✓	✓	✓	✓
DNA helicase	13420	14685	1266	forward	ALP47373	✓	✓	✓	✓
Nicotinamide-nucleotide adenylyltransferase NadM family/ADP-ribose pyrophosphatase	17192	18217	1026	forward	ALP47387	✓	✓	✓	✓
Nicotinate nucleotide adenylyltransferase	18965	19501	537	forward	ALP47387	✓	✓	✓	✓
Thymidine kinase	30891	31460	570	forward	ALP47460	✓	✓		✓
Endonuclease V	38697	39101	405	forward	ALP47535	✓	✓	✓	✓
Nicotinamide-nucleotide adenylyltransferase NadR family/Ribosylnicotinamide kinase	41381	42361	981	forward	ALP47393	✓	✓	✓	✓
Ribosyl nicotinamide transporter	43313	43987	675	forward	ALP47436	✓	✓	✓	✓
Nicotinamide phosphoribosyltransferase	59435	60928	1494	forward	ALP47363	✓	✓	✓	✓
Ribonucleoside-diphosphate reductase alpha subunit	65364	67589	2226	forward	ALP47345	✓	✓	✓	✓
Ribonucleoside-diphosphate reductase beta subunit	67599	68723	1125	forward	ALP47379	✓	✓	✓	✓
Thioredoxin	68725	69024	300	forward	ALP47594	✓	✓	✓	✓
Transglycosylase	69098	69670	573	forward	ALP47458	✓	✓	✓	✓
Thioredoxin	69957	70961	1005	forward	ALP47391	✓	✓	✓	✓
DNA topoisomerase	71007	72293	1287	forward	ALP47370	✓	✓	✓	✓
Queuosine biosynthesis QueE radical SAM	72544	73428	885	forward	ALP47407	✓	✓	✓	✓
Putative ATPase	76549	77253	705	forward	ALP47429	✓	✓	✓	✓
Anti-sigma factor	78533	78832	300	forward	ALP47593	✓	✓	✓	✓
Tail fibers protein	79799	83812	4014	reverse	ALP47337	✓	✓	✓	
Tail fibers protein	83884	85614	1731	reverse	ALP47353	✓	✓	✓	
Tail fibers protein	89115	90650	1536	reverse	ALP47361	✓	✓	✓	
Deoxynucleotide monophosphate kinase	107670	108314	645	reverse	ALP47441	✓	✓	✓	
Tail completion protein	108544	109080	537	reverse	ALP47471	✓	✓	✓	✓
Putative baseplate hub catalyst	111053	111220	168	reverse	ALP47703	✓	✓	✓	
Baseplate hub assembly chaperone	111220	112068	849	reverse	ALP47411	✓	✓	✓	✓
Baseplate tail tube initiator	112081	112827	747	reverse	ALP47421	✓	✓	✓	✓
DNA end protector during packaging	112831	113427	597	reverse	ALP47449		✓	✓	

Supplemental Table 3. Gene functions of the *Vibrio* phage φSt2 *(Continue)*

Head completion protein	113429	113884	456	reverse	ALP47512	✓	✓	✓	
Baseplate tail tube cap	113952	115088	1137	forward	ALP47378	✓	✓	✓	
Baseplate wedge subunit	115085	115663	579	forward	ALP47455	✓	✓	✓	✓
Baseplate hub subunit/ Tail lysozyme	116942	118153	1212	forward	ALP47375	✓	✓	✓	✓
Phospholipase	118643	118939	297	forward	ALP47599	✓	✓	✓	✓
Baseplate wedge subunit	120353	120772	420	forward	ALP47527	✓	✓	✓	✓
Baseplate wedge subunit	120858	122816	1959	forward	ALP47349	✓	✓	✓	✓
Baseplate wedge subunit	122816	126313	3498	forward	ALP47339	✓	✓	✓	✓
Baseplate wedge subunit	126315	127337	1023	forward	ALP47388	✓	✓	✓	✓
Baseplate wedge tail fiber connector	127391	128347	957	forward	ALP47394	✓	✓	✓	✓
Baseplate wedge subunit and tail pin	128357	130603	2247	forward	ALP47343	✓	✓	✓	✓
Baseplate wedge subunit and tail pin	130603	131289	687	forward	ALP47435	✓	✓	✓	✓
Straight tail fiber	131289	132827	1539	forward	ALP47360	✓	✓	✓	✓
Tail fiber protein	132824	134251	1428	forward	ALP47360	✓	✓	✓	✓
Neck whiskers protein	134551	136233	1683	forward	ALP47355	✓	✓	✓	✓
Head completion neck hetero-dimeric protein	136244	137173	930	forward	ALP47398	✓	✓	✓	✓
Head completion neck hetero-dimeric protein	137177	138016	840	forward	ALP47413	✓	✓	✓	✓
Tail assembly protein	138026	139087	1062	forward	ALP47383	✓	✓	✓	✓
Terminase small subunit	139799	140347	549	forward	ALP47467	✓	✓	✓	✓
Terminase large subunit	140307	142109	1803	forward	ALP47351	✓	✓	✓	✓
Tail sheath monomer	142156	144171	2016	forward	ALP47347	✓	✓	✓	✓
Tail tube monomer	144223	144723	501	forward	ALP47486	✓	✓	✓	✓
Portal vertex of the head	144763	146313	1551	forward	ALP47358	✓	✓	✓	✓
Prohead core protein	146327	146494	168	forward	ALP47702	✓	✓	✓	✓
Capsid and scaffold	146495	146986	492	forward	ALP47491	✓	✓	✓	✓
Prohead assembly (scaffolding) protein	146989	147630	642	forward	ALP47442	✓	✓	✓	✓
Prohead assembly (scaffolding) protein	147663	148511	849	forward	ALP47410	✓	✓	✓	✓
Major capsid protein	148582	150126	1545	forward	ALP47359	✓	✓	✓	✓
tRNA nucleotidyltransferase	150184	151281	1098	reverse	ALP47382		✓	✓	
Inhibitor of prohead protease	151377	151874	498	forward	ALP47488	✓	✓	✓	✓
DNA helicase	158008	159537	1530	forward	ALP47362	✓	✓	✓	✓
Transamidase GatB domain protein	160023	160436	414	reverse	ALP47530	✓	✓	✓	
Single stranded DNA-binding protein	160433	160846	414	reverse	ALP47531	✓	✓	✓	✓

Supplemental Table 3. Gene functions of the *Vibrio* phage φSt2 *(Continue)*

Tail connector protein	161015	163708	2694	reverse	ALP47341	✓	✓	✓	
Short tail fiber protein	163717	167424	3708	reverse	ALP47338	✓	✓	✓	
Ribonuclease H	167503	168435	933	forward	ALP47396	✓	✓	✓	✓
Double-stranded DNA binding protein	168518	168799	282	forward	ALP47611	✓	✓	✓	✓
Transcriptional regulator	168783	169085	303	forward	ALP47590	✓	✓		✓
DNA helicase loader	169120	169680	561	forward	ALP47462	✓	✓	✓	✓
Single stranded DNA-binding protein	169731	170657	927	forward	ALP47399	✓	✓	✓	✓
Dihydrofolate reductase	170707	171252	546	forward	ALP47469	✓	✓	✓	✓
ATP-dependent Clp protease proteolytic subunit	171249	171965	717	forward	ALP47427	✓	✓	✓	✓
Recombination protein	172032	173132	1101	forward	ALP47381	✓	✓	✓	✓
Head assembly protein	173187	173489	303	forward	ALP47589	✓	✓	✓	✓
DNA primase/helicase	173543	174826	1284	forward	ALP47371	✓	✓	✓	✓
Ribonucleoside-triphosphate reductase	175066	176901	1836	forward	ALP47350	✓	✓	✓	✓
Ribonucleoside-triphosphate reductase activating protein	178022	178498	477	forward	ALP47498	✓	✓	✓	✓
Phosphoesterase	178498	179022	525	forward	ALP47473	✓	✓	✓	✓
DNA helicase	179936	180760	825	forward	ALP47415	✓	✓	✓	✓
DNA primase/ DNA helicase	181319	182377	1059	forward	ALP47384	✓	✓	✓	✓
Deoxyuridine 5'-triphosphate nucleotidohydrolase	182377	182874	498	forward	ALP47489	✓	✓	✓	✓
DexA exonuclease A	183107	183799	693	forward	ALP47434	✓	✓	✓	✓
Thymidylate synthase	185703	186602	900	forward	ALP47405	✓	✓	✓	✓
**NAD-dependent protein deacetylase of SIR2/cobB family**	**190808**	**191569**	**762**	**forward**	**ALP47418**	✓	✓	✓	✓
Topoisomerase IV subunit B	191704	193497	1794	forward	ALP47352	✓	✓	✓	✓
Ser/Thr protein phosphatase family protein	194881	195609	729	forward	ALP47424	✓	✓	✓	
DNA ligase	198013	199350	1338	forward	ALP47369	✓	✓	✓	✓
RNA polymerase-ADP-ribosyltransferase Alt	200334	201932	1599	reverse	ALP47357	✓	✓	✓	
Glutaredoxin	203720	203959	240	forward	ALP47647	✓	✓	✓	✓
Capsid vertex protein	204015	204914	900	forward	ALP47404	✓	✓	✓	✓
RNA polymerase sigma factor for late transcription	204923	205435	513	forward	ALP47478	✓	✓	✓	✓
Putative 5'(3')-deoxyribonucleotidase	209207	209707	501	forward	ALP47487	✓	✓	✓	✓
Recombination-related endonuclease	209704	210747	1044	forward	ALP47386	✓	✓	✓	✓
Recombination-related endonuclease	210964	213201	2238	forward	ALP47344	✓	✓	✓	✓
Sliding clamp DNA polymerase accessory protein	213963	214628	666	forward	ALP47438	✓	✓	✓	✓

Supplemental Table 3. Gene functions of the *Vibrio* phage φSt2 *(Continue)*

Replication factor C small subunit /DNA polymerase clamp loader subunit	214695	215645	951	forward	ALP47395	✓	✓	✓	✓
DNA polymerase clamp loader subunit	215655	216146	492	forward	ALP47492	✓	✓	✓	✓
Homing endonuclease (Seg-like)	***216143***	***216727***	***585***	***reverse***	***ALP47453***				
Endoribonulcease translational repressor of early genes regA	216793	217176	384	forward	ALP47542	✓		✓	✓
DNA polymerase	218252	220804	2553	forward	ALP47342	✓	✓	✓	✓
RNA ligase A	221127	222272	1146	forward	ALP47377	✓	✓	✓	✓
Beta lactamase domain protein	223668	223901	234	forward	ALP47653	✓	✓	✓	✓
3'-phosphatase 5'-polynucleotide kinase	223910	224827	918	forward	ALP47402	✓	✓	✓	✓
DCMP deaminase	238156	238608	453	forward	ALP47515	✓	✓		✓
NADPH-dependent 7-cyano-7-deazaguanine reductase	238663	239595	933	forward	ALP47397	✓	✓		✓
GTP cyclohydrolase I	239663	240337	675	forward	ALP47437	✓	✓	✓	✓
Homing endonuclease (mob-like)	***240309***	***241010***	***702***	***reverse***	***ALP47430***		✓		
DNA methyltransferase	244175	244678	504	forward	ALP47484				
NADPH dependent preQ0 reductase	244662	245573	912	forward	ALP47403	✓	✓	✓	✓
Queuosine Biosynthesis QueC ATPase	245629	246345	717	forward	ALP47426	✓	✓	✓	✓
Head assembly chaperone protein	247742	248080	339	forward	ALP47563	✓	✓	✓	✓
Endonuclease	248502	248930	429	reverse	ALP47524	✓	✓	✓	
RNA ligase	249352	250359	1008	forward	ALP47390	✓	✓	✓	✓
