# Supplementary material for: Comparative Functional Genomic Analysis of Two Vibrio Phages Reveals Complex Metabolic Interactions with the Host Cell
Source: Front Microbiol. 2016 Nov 14;7:1807. doi: 10.3389/fmicb.2016.01807 (PMC5107563; doi:10.3389/fmicb.2016.01807)
Supplement: Supplementary file 4 [file Data_Sheet_1.DOCX]

**Supplemental Data Sheet 1**

**Materials and Methods**

## Amplification, precipitation and DNA extraction of bacteriophages

Two liquid bacterial cultures of *Vibrio alginolyticus* strain V1 in the exponential phase of growth were infected separately by bacteriophages *φ*St2 and *φ*Grn1. The infection was performed with a multiplicity of infection (MOI) of 10 and both tubes were incubated overnight at 25°C with reciprocal shaking. The following day, the cultures were centrifuged and their supernatants were filtered (0.22 μm), tittered and stored at 4°C. Phage lysate was filtered and bacteriophages *φ*Grn1 and *φ*St2 were stored at 4^o^C. Having an optimal titer of 10^10^ PFU ml^-1^, phages were concentrated using a standard poly-ethylene glycol/NaCl precipitation. Specifically, a stock of 20% polyethylene glycol with 2.5M NaCl was prepared and 400μl were added in 1.6ml of phage stock to a final volume of 2 ml. The mixture was left overnight at 4^o^C and a 30 min centrifugation at 13.500 rpm was performed at 4^o^C. The supernatant was discarded and the pellet was suspended in 100 μl of 150 mM NaCl. Two precipitations for each phage were mixed and incubated with RNase and DNase to remove host’s nucleic acids. DNA extraction was conducted using a Qiagen protocol of the QIAamp DNA Blood Mini Kit (QIAGEN, Hilden, Germany) with the addition of ethanol 100% before the first column wash. A yield of at least 10 μg of DNA was retrieved. Finally, PCR with 16S universal primers (Forward: 5’-AGAGTTTGATCCTGGCTCAG-3’, Reverse: 5’-GACGGGCGGTGTGTACAAG-3’) was conducted before and after DNase treatment in order to verify the absence of the host’s or other contaminant DNA [95, 96]. DNA quality was evaluated with NanoDrop (Thermo Fisher Scientific, Waltham, MA, USA) measurements and agarose gel before library construction.

**Transcriptional study of bacterial and viral genes**

Gene expression was studied in wild type (uninfected control) and phage-treated bacteria (MOI:100). Bacteriophages were incorporated into bacterial cultures during exponential phase. Three biological replicates were used for both treatments. After 1 min of vigorous shaking at 25 ^o^C, 5ml of each phage-treated culture were harvested. Harvest was repeated at 5, 10, 20 and 30 min post infection (p.i.). For the control treatments only one harvest per replicate was performed at 30 min. Cells were immediately centrifuged at 4 ^o^C and washed with 150mM NaCl prior to RNA extraction. The duration of the experiment was set based on the latency time which is 30 min for both phages [18].

RNA extraction was performed using a standard TRIzol^TM^ (Thermo Fisher Scientific, Waltham, MA, USA) protocol. Specifically, after harvesting, the cells were homogenized in 500μl TRIzol^TM^ and left overnight at -20^o^C. Bacterial and viral RNA was then isolated with chloroform and precipitated with isopropanol. Finally, the RNA was washed with 75% of Ethanol and suspend in ddH_2_O. This method resulted in at least 13μg of RNA per sample.

6μg of RNA per sample were treated with DNase RQ1 (Promega, Madison, WI, USA) according to manufacturer’s protocol. Samples were tested with PCR to verify purity from bacterial and viral DNA. RNA was then extracted by using a phenol: chloroform protocol. A 70% yield of RNA was retrieved after DNase treatment. RNA quality was also examined by electrophoresis to avoid using a hydrolyzed sample for cDNA synthesis. Approximately 1μg of RNA was used per cDNA synthesis by using Superscript II (Thermo Fisher Scientific, Waltham, MA, USA) enzyme. Specifically, first-strand cDNA was reverse transcribed from 1μg of RNA, the samples were denatured at 65^o^C for 5 min followed by quick cooling on ice in a 12μl reaction mixture containing 10μl of RNA, 1μl of random primers (3μg/ml) and 1μl of 10mM dNTPs. After the addition of 4 μl of 5x First-Strand buffer (Thermo Fisher Scientific, Waltham, MA, USA), 1μl (40 U) of RNaseOUT (Thermo Fisher Scientific, Waltham, MA, USA) RNase inhibitor and 2 μl of 0.1M dithiothreitol (DTT), the reaction was incubated at 25^o^C for 2 min before the addition of 1 μl (200 U) of SuperScript II reverse transcriptase. The reaction was incubated again at 25^o^C for an additional 10 min, followed by incubation at 42^o^C for 50 min and a final heat-inactivation at 70^o^C for 15 min. cDNAs were then stored at -20^o^C.

Both bacterial and viral primers for cDNA amplification were designed using Geneious software **(Supplemental Table 1)** and were tested against both bacterial and bacteriophage genomic DNA to confirm that a single amplicon of 70 base pairs would result from qPCR. Quantitative real time PCR was performed on a StepOnePlus™ Real-Time PCR System (Applied Biosystems, Foster City, CA, USA) using SYBR Select Master Mix (Applied Biosystems, Austin, TX, USA), gene-specific primers at a final concentration of 0.2 μΜ each, and 1 μl of the cDNA as template. PCR cycling started with the initial polymerase activation at 95^o^C for 10 min, followed by 40 cycles at 95^o^C for 15 seconds and 60^o^C for 1 min. Primer specificity and formation of primer dimers were monitored by dissociation curve analysis. The expression levels of *V. alginolyticus* gyrase A (*gyrA*) and the HSP70 protein (*dnaK*) were used as housekeeping genes (HK) to normalize cDNA templates. Relative transcript levels of the gene of interest were calculated as primer Efficiency^-ΔCt^, where ΔCt is the difference between the geometrical mean of the two HK genes’ Cts and the Ct of the gene of interest, while primer efficiency is the mean of each primer’s efficiency, which was calculated by employing the linear regression method on the Log (Fluorescence) per cycle number data (ΔRn), using the LinRegPCR software (Ramakers et al., 2003). In order to evaluate the experiment’s and the HK gene’s reliability, 2 viral gene expression motifs were studied at first after normalization. The first one is a Glutaredoxin gene (*grx*) which is considered as a late early transcribed phage gene and the second one the major capsid protein (*MCP*), which is considered as a late transcribed one **(Supplemental Figure 1)** [98].

**References**

Ramakers C, Ruijter JM, Lekanne Deprez RH, Moorman AFM. (2003). Assumption-free analysis of quantitative real-time polymerase chain reaction (PCR) data. *Neurosci. Lett.* 339, 62–66.
