# Supplementary material for: Comparative Functional Genomic Analysis of Two Vibrio Phages Reveals Complex Metabolic Interactions with the Host Cell
Source: Front Microbiol. 2016 Nov 14;7:1807. doi: 10.3389/fmicb.2016.01807 (PMC5107563; doi:10.3389/fmicb.2016.01807)
Supplement: Supplementary file 5 [file Data_Sheet_2.PDF]

Data Sheet 2. Relative transcript values (± SE) of *V. alginolyticus* strain V1 (A) and φSt2 (B) genes. Corresponding heat maps shown in Figure 5 are highlighted.

A.

Treatment	Gene	Relative Expression	SE	Gene	Relative Expression	SE	Gene	Relative Expression	SE	Gene	Relative Expression	SE
Control	*nrdB*	0.677035225	0.07997	*nrD1*	0.004257074	0.00414	*nrD2*	0.004140938	0.001221	*NDK*	0.1302	5.04E-03
1 minute p.i.	*nrdB*	0.343469531	0.05396	*nrD1*	0.005435401	0.00799	*nrD2*	0.007989359	0.002182	*NDK*	0.0919	4.51E-03
5 minutes p.i.	*nrdB*	0.364827572	0.06413	*nrD1*	0.007543919	0.01633	*nrD2*	0.016331852	0.002769	*NDK*	0.1164	5.90E-03
10 minutes p.i.	*nrdB*	0.4144773	0.0651	*nrD1*	0.004758148	0.0115	*nrD2*	0.011497455	0.001435	*NDK*	0.1618	8.48E-03
20 minutes p.i.	*nrdB*	0.392136624	0.0406	*nrD1*	0.003978105	0.01649	*nrD2*	0.016486242	0.008984	*NDK*	0.1677	1.48E-03
30 minutes p.i.	*nrdB*	0.385304088	0.14161	*nrD1*	0.026040271	0.03666	*nrD2*	0.036663304	0.020038	*NDK*	0.1121	6.32E-03

Treatment	Gene	Relative Expression	SE	Gene	Relative Expression	SE	Gene	Relative Expression	SE	Gene	Relative Expression	SE
Control	*ACS*	5.62E-03	4.76E-04	*Sir2*	0.0133	1.15E-03	*NMNAT*	0.0419	2.91E-04	*pncA*	0.0546	6.30E-03
1 minute p.i.	*ACS*	6.38E-03	4.66E-04	*Sir2*	0.0139	1.70E-03	*NMNAT*	0.0478	4.63E-03	*pncA*	0.0643	8.88E-03
5 minutes p.i.	*ACS*	4.10E-03	6.82E-04	*Sir2*	0.0114	2.88E-04	*NMNAT*	0.0407	5.24E-03	*pncA*	0.0603	5.34E-03
10 minutes p.i.	*ACS*	4.89E-03	3.95E-04	*Sir2*	0.0186	1.94E-03	*NMNAT*	0.0473	5.31E-03	*pncA*	0.0814	7.56E-03
20 minutes p.i.	*ACS*	4.58E-03	6.01E-04	*Sir2*	0.0238	1.20E-03	*NMNAT*	0.0464	2.46E-03	*pncA*	0.0698	4.61E-03
30 minutes p.i.	*ACS*	0.0135	1.17E-03	*Sir2*	0.0315	1.20E-03	*NMNAT*	0.0412	5.43E-03	*pncA*	0.0584	3.79E-03

Treatment	Gene	Relative Expression	SE	Gene	Relative Expression	SE	Gene	Relative Expression	SE
Control	*TMK*	0.1574	0.0214	*TYMS1*	0.311510625	0.08019	*TYMS2*	0.016288251	0.00263
1 minute p.i.	*TMK*	0.0726	0.0156	*TYMS1*	0.171735011	0.04667	*TYMS2*	0.008227093	0.00422
5 minutes p.i.	*TMK*	0.1206	4.40E-03	*TYMS1*	0.212795398	0.01678	*TYMS2*	0.014604793	0.00343
10 minutes p.i.	*TMK*	0.0886	5.35E-03	*TYMS1*	0.221664245	0.02848	*TYMS2*	0.019071085	0.00285
20 minutes p.i.	*TMK*	0.1323	0.017	*TYMS1*	0.235064069	0.02066	*TYMS2*	0.023821018	0.00285
30 minutes p.i.	*TMK*	0.1638	0.0186	*TYMS1*	0.430320391	0.1137	*TYMS2*	0.03426018	0.00831

B.

Treatment	Gene	Relative expression	SE	Gene	Relative expression	SE
1 minute p.i.	*MCP*	0.3723	0.1656	*DUT*	0.1527	2.84E-03
5 minutes p.i.	*MCP*	3.0162	0.4396	*DUT*	1.3397	0.1042
10 minutes p.i.	*MCP*	4.9353	1.0555	*DUT*	9.046	0.6725
20 minutes p.i.	*MCP*	17.595	2.703	*DUT*	34.0174	5.3015
30 minutes p.i.	*MCP*	94.3912	14.5341	*DUT*	15.5621	2.3077
**Treatment**	**Gene**	**Relative expression**	**SE**	**Gene**	**Relative expression**	**SE**
1 minute p.i.	*grx*	0.0964	1.81E-03	*TYMS*	0.1196	1.86E-03
5 minutes p.i.	*grx*	0.4875	0.0595	*TYMS*	6.5248	1.5237
10 minutes p.i.	*grx*	12.5785	3.5296	*TYMS*	23.1372	7.8951
20 minutes p.i.	*grx*	8.828	1	*TYMS*	29.1679	8.4112
30 minutes p.i.	*grx*	5.3073	0.7948	*TYMS*	8.4729	1.8125
**Treatment**	**Gene**	**Relative expression**	**SE**	**Gene**	**Relative expression**	**SE**
1 minute p.i.	*NAMPT*	0.4073	9.48E-03	*Sir2*	0.0657	0.0124
5 minutes p.i.	*NAMPT*	14.1438	2.4144	*Sir2*	4.3085	0.2549
10 minutes p.i.	*NAMPT*	27.8278	5.9351	*Sir2*	4.9353	0.5988
20 minutes p.i.	*NAMPT*	67.6817	14.0995	*Sir2*	12.163	1.4249
30 minutes p.i.	*NAMPT*	52.5689	4.062	*Sir2*	3.3882	0.5581
**Treatment**	**Gene**	**Relative expression**	**SE**	**Gene**	**Relative expression**	**SE**
1 minute p.i.	*NMNAT*	0.2117	0.0877	*nrdB*	0.1336	8.29E-03
5 minutes p.i.	*NMNAT*	4.9885	0.3742	*nrdB*	0.8561	0.0431
10 minutes p.i.	*NMNAT*	16.0469	3.773	*nrdB*	9.4089	0.6354
20 minutes p.i.	*NMNAT*	12.5093	2.3824	*nrdB*	17.7425	2.77E+00
30 minutes p.i.	*NMNAT*	10.9091	1.4877	*nrdB*	27.6266	3.7077
