# Supplementary material for: Comparative Functional Genomic Analysis of Two Vibrio Phages Reveals Complex Metabolic Interactions with the Host Cell
Source: Front Microbiol. 2016 Nov 14;7:1807. doi: 10.3389/fmicb.2016.01807 (PMC5107563; doi:10.3389/fmicb.2016.01807)
Supplement: Supplementary file 6 [file Data_Sheet_3.DOCX]

Supplementary Figures

Comparative functional genomic analysis of two *Vibrio* phages reveals complex metabolic interactions with the host cell

**Dimitrios Skliros^1^, Panos G. Kalatzis^2, 3^, Pantelis Katharios^2^, Emmanouil Flemetakis^1^***

^1^ Laboratory of Molecular Biology, Department of Biotechnology, School of Food, Biotechnology and Development, Agricultural University of Athens

^2^ Institute of Marine Biology, Biotechnology and Aquaculture, Hellenic Centre for Marine Research, Heraklion, Crete, Greece

^3^ Marine Biological Section, University of Copenhagen, Helsingør, Denmark

*** Correspondence:**Emmanouil Flemetakis, Laboratory of Molecular Biology, Department of Biotechnology, School of Food, Biotechnology and Development, Agricultural University of Athens, Iera odos, 75, street, 11855, Attika, Athens, Greece.
[mflem@aua.gr](mailto:mflem@aua.gr)

5 Supplementary Figures.

## Supplementary Figures


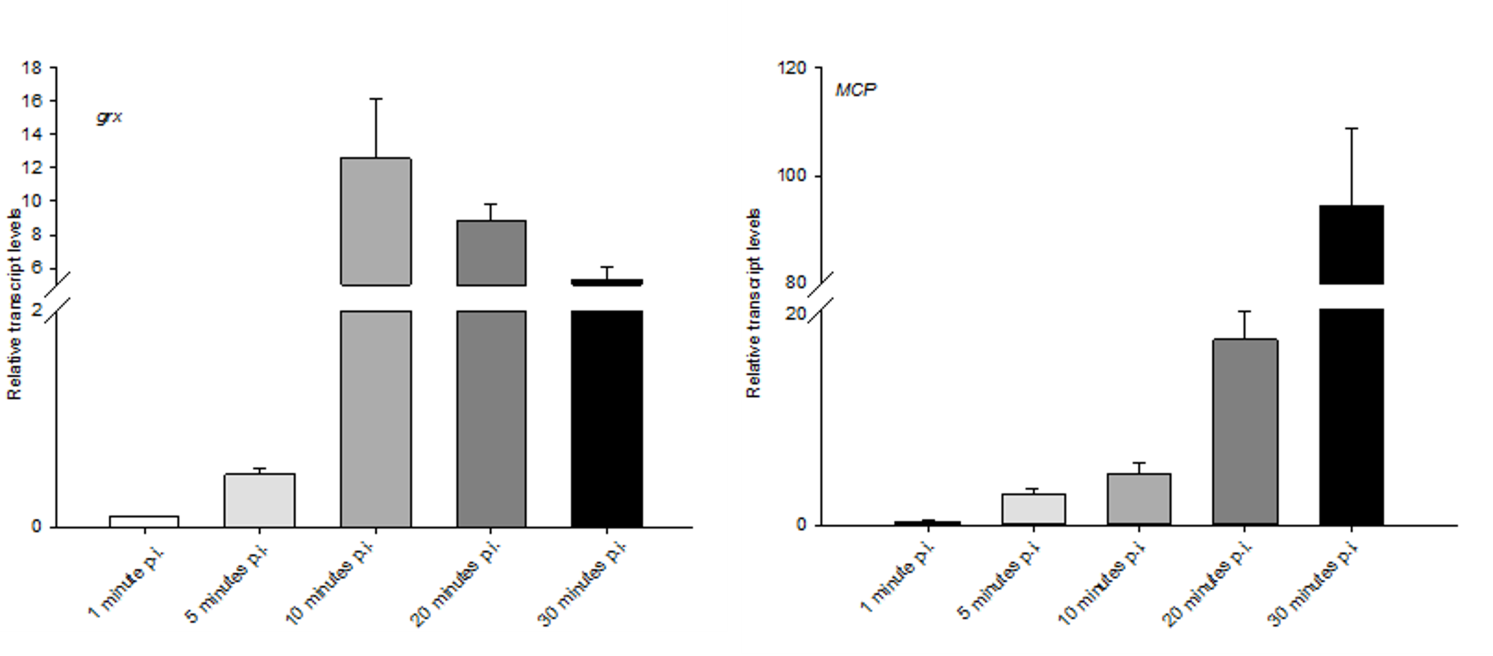


**Supplementary Figure 1.** **Relative transcript levels of two bacteriophage genes.** Relative transcript levels (± SE) of *MCP* (major capsid protein) and *grx* (glutaredoxin protein) genes during infection are represented by bars. *Grx* gene is a delayed early transcribed gene in T4-like phages, while *MCP* is a late transcribed gene, during phage infection.


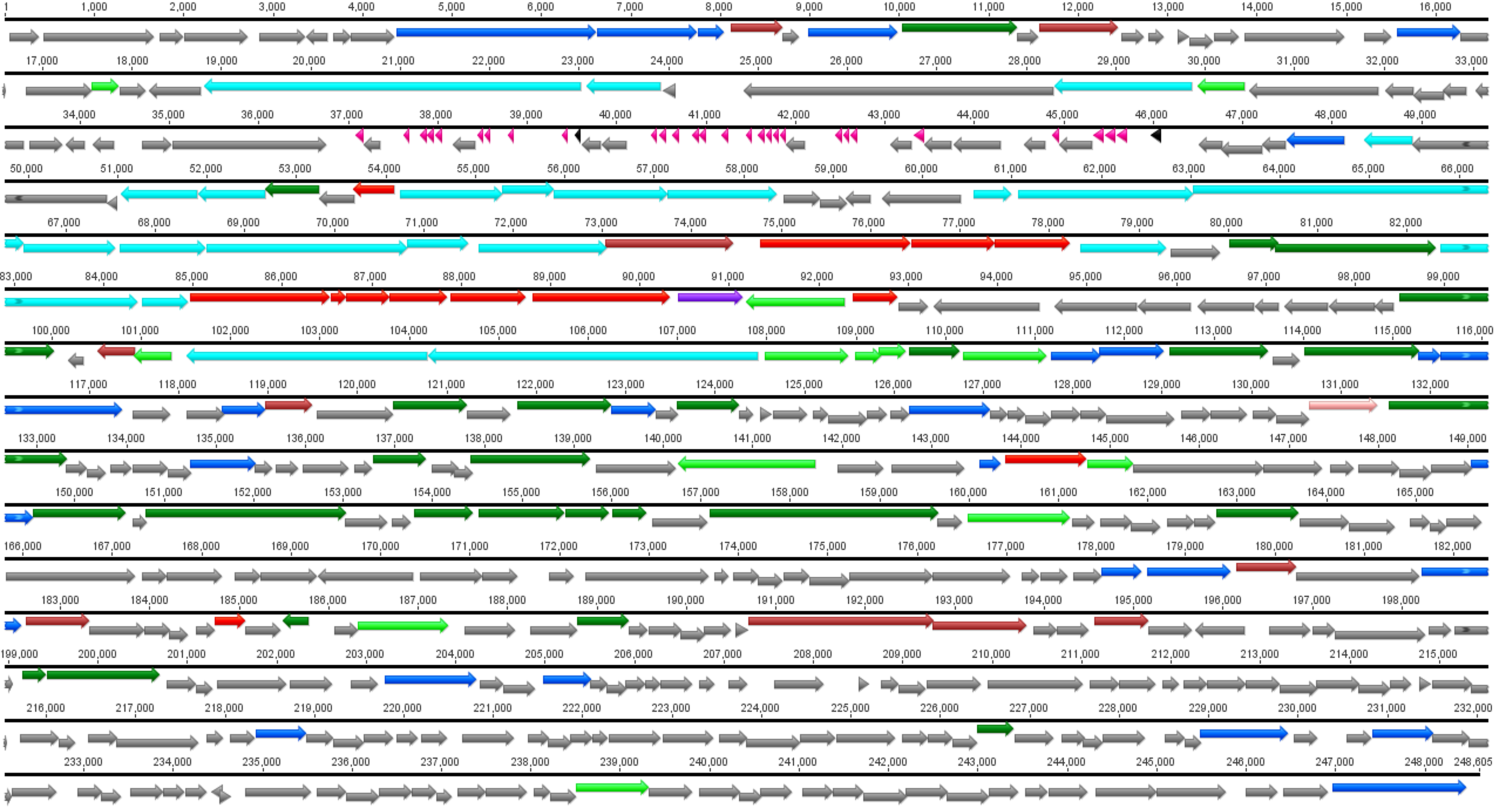


**Supplementary Figure 2.** **Genomic map of *φ*Grn1 bacteriophage.** Arrows represent annotated CDSs strand orientation. Colors: Dark green 🡪DNA replication and packaging, Light green 🡪 RNA metabolism, Light blue🡪 Tail structure, associated enzymes of baseplate and fibers, Red 🡪Capsid proteins, Dark blue 🡪 NAD^+^ and Nucleotides metabolism related, Pink🡪 Sir2/cobB, Brown 🡪 Others, Grey🡪 Hypothetical proteins, Fuchsia🡪 Verified transfer RNAs, Black🡪 Pseudo transfer RNAs.


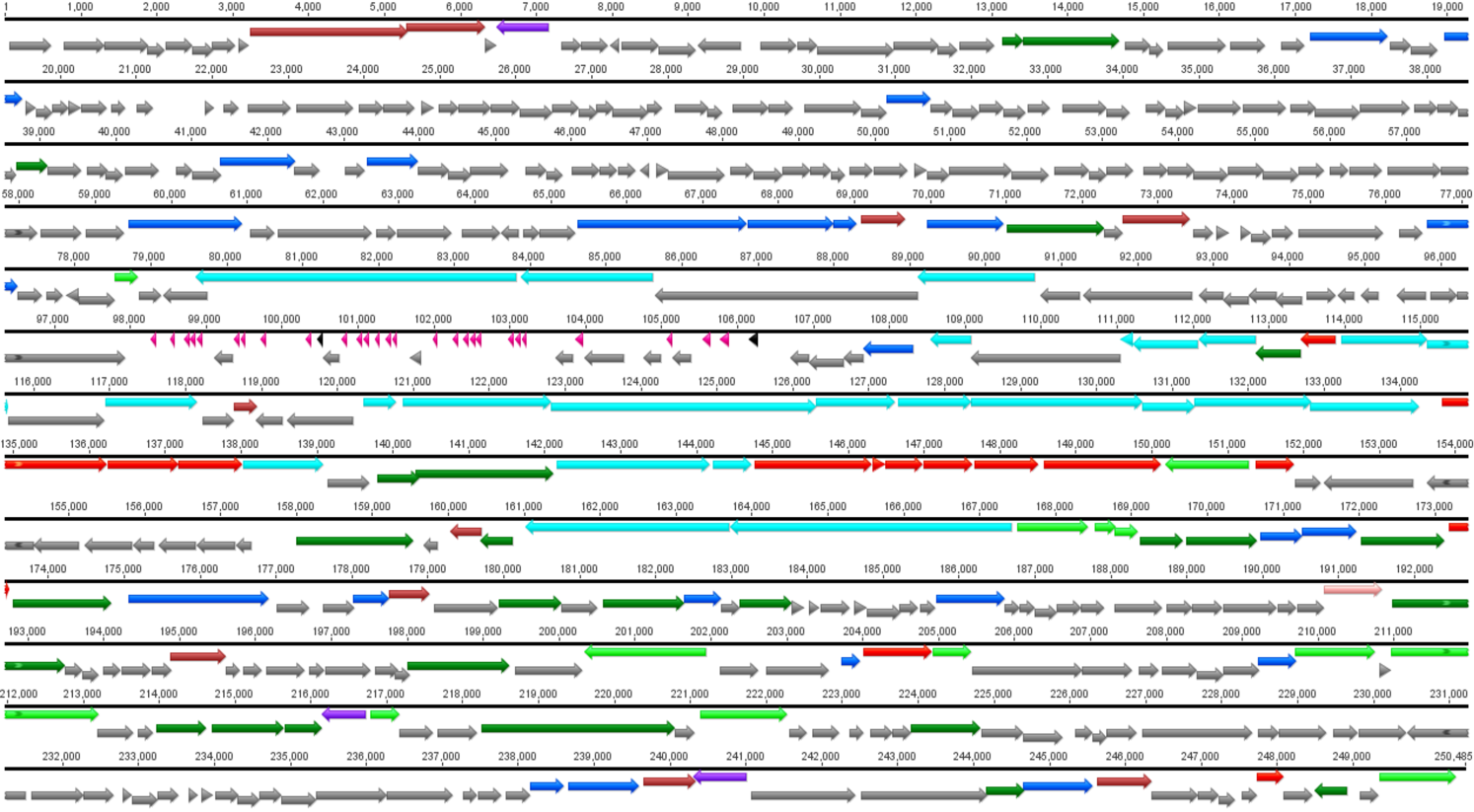


**Supplementary Figure 3.** **Genomic map of *φ*St2 bacteriophage.** Arrows represent annotated CDSs strand orientation. Colors: Dark green 🡪DNA replication and packaging, Light green 🡪 RNA metabolism, Light blue🡪 Tail structure, associated enzymes of baseplate and fibers, Red 🡪Capsid proteins, Dark blue 🡪 NAD^+^ and Nucleotides metabolism related, Pink🡪 Sir2/cobB, Brown 🡪 Others, Grey🡪 Hypothetical proteins, Fuchsia🡪 Verified transfer RNAs, Black🡪 Pseudo transfer RNAs.


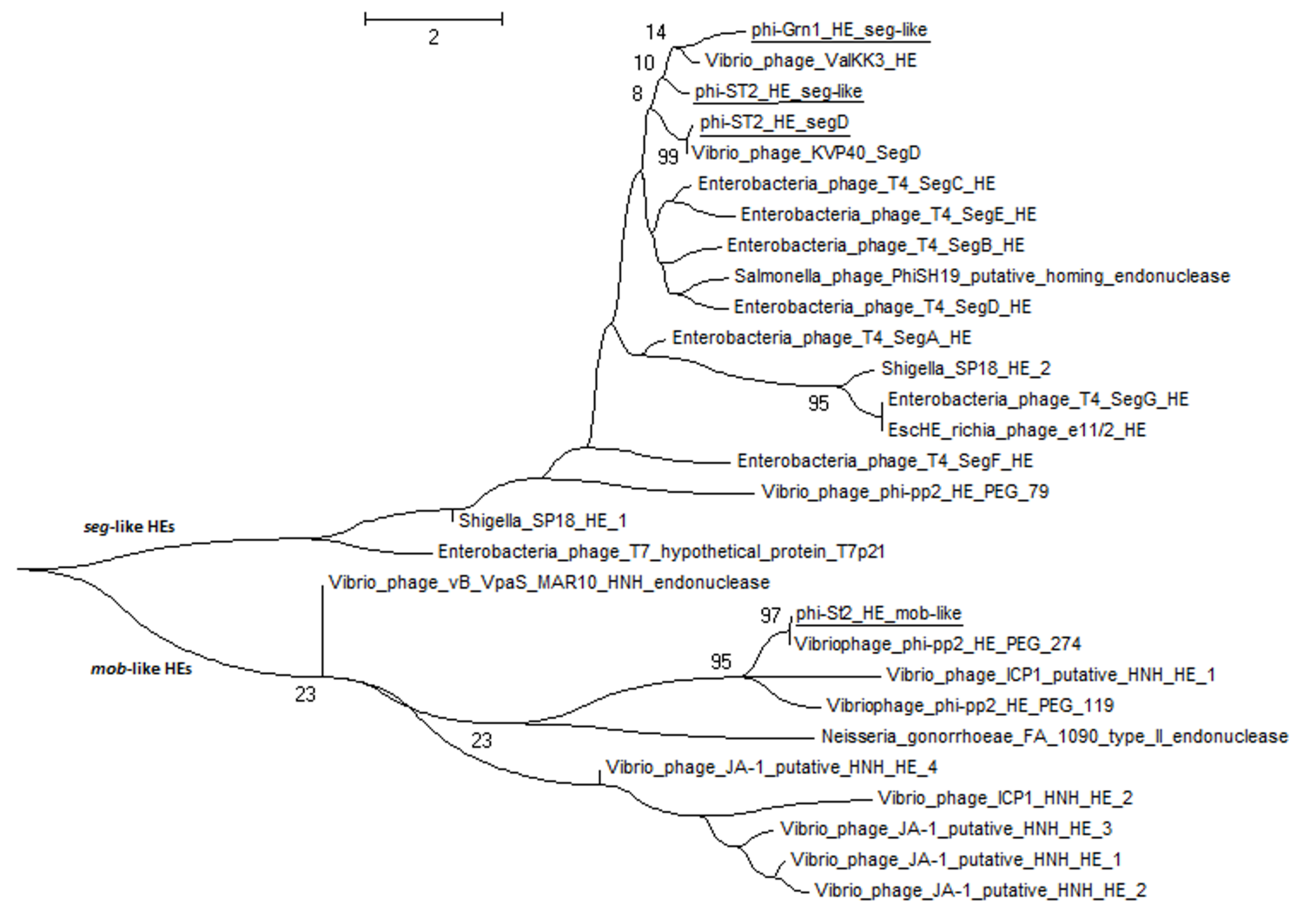


**Supplementary Figure 4.** **Maximum likelihood tree between various phage-associated homing** endonucleases. Numbers on branches represent relative likelihood support scores (RLS). Only important RLSs are depicted. Homing endonucleases of *φ*Grn1 and *φ*St2 bacteriophages are underlined.


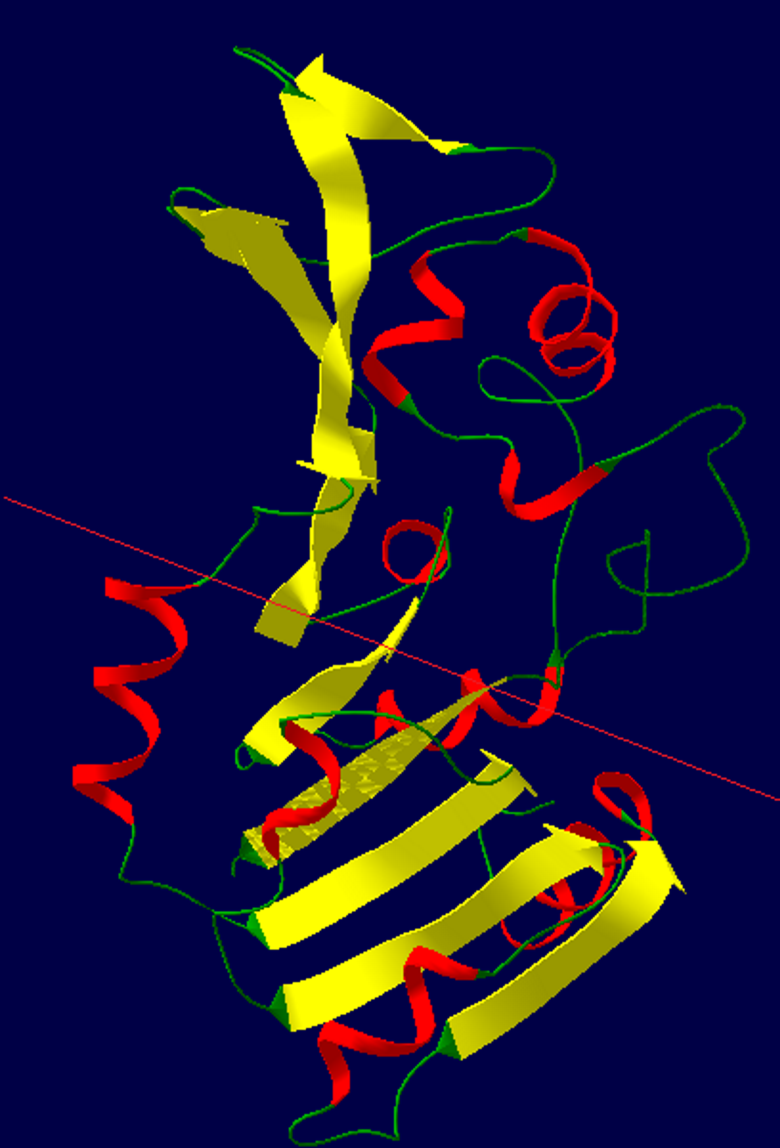


**Supplementary Figure 5. Schematic representation of *φ*St2 and *φ*Grn1 Sir2/cobB protein.** Ribbon diagram of the *φ*St2 Sir2/cobB protein. Sheets (yellow), helices (red), and coils (green) are highlighted. The large Rossman domain (down) is separated from the active site and small finger domain (up) by a red line.
